# Molecular and Cellular Characterization of a Zebrafish Optic Pathway Tumor Line Implicates Glia-Derived Progenitors in Tumorigenesis

**DOI:** 10.1371/journal.pone.0114888

**Published:** 2014-12-08

**Authors:** Staci L. Solin, Ying Wang, Joshua Mauldin, Laura E. Schultz, Deborah E. Lincow, Pavel A. Brodskiy, Crystal A. Jones, Judith Syrkin-Nikolau, Jasmine M. Linn, Jeffrey J. Essner, Jesse M. Hostetter, Elizabeth M. Whitley, J. Douglas Cameron, Hui-Hsien Chou, Andrew J. Severin, Donald S. Sakaguchi, Maura McGrail

**Affiliations:** 1 Department of Genetics, Development and Cell Biology, Iowa State University, Ames, Iowa, United States of America; 2 Department of Veterinary Pathology, Iowa State University, Ames, Iowa, United States of America; 3 Department of Ophthalmology and Visual Neurosciences, University of Minnesota, Minneapolis, Minnesota, United States of America; 4 Genome Informatics Facility, Office of Biotechnology, Iowa State University, Ames, Iowa, United States of America; University of Pittsburgh School of Medicine, United States of America

## Abstract

In this study we describe the molecular and cellular characterization of a zebrafish mutant that develops tumors in the optic pathway. Heterozygous *Tg(flk1:RFP)is18* transgenic adults develop tumors of the retina, optic nerve and optic tract. Molecular and genetic mapping demonstrate the tumor phenotype is linked to a high copy number transgene array integrated in the lincRNA gene *lincRNAis18/Zv9_00007276* on chromosome 3. TALENs were used to isolate a 147kb deletion allele that removes exons 2–5 of the *lincRNAis18* gene. Deletion allele homozygotes are viable and do not develop tumors, indicating loss of function of the *lincRNAis18* locus is not the trigger for tumor onset. Optic pathway tumors in the *Tg(flk1:RFP)is18* mutant occur with a penetrance of 80–100% by 1 year of age. The retinal tumors are highly vascularized and composed of rosettes of various sizes embedded in a fibrous matrix. Immunohistochemical analysis showed increased expression of the glial markers GFAP and BLBP throughout retinal tumors and in dysplastic optic nerve. We performed transcriptome analysis of pre-tumorous retina and retinal tumor tissue and found changes in gene expression signatures of radial glia and astrocytes (*slc1a3*), activated glia (*atf3, blbp, apoeb*), proliferating neural progenitors (*foxd3, nestin, cdh2, her9/hes1*), and glioma markers (*S100β, vim*). The transcriptome also revealed activation of cAMP, Stat3 and Wnt signal transduction pathways. qRT-PCR confirmed >10-fold overexpression of the Wnt pathway components *hbegfa*, *ascl1a*, and *insm1a*. Together the data indicate Müller glia and/or astrocyte-derived progenitors could contribute to the zebrafish *Tg(flk1:RFP)is18* optic pathway tumors.

## Introduction

Glia play critical roles in the function and maintenance of the nervous system. They are involved in neuronal homeostasis and repair, but can also undergo reprogramming in response to injury to generate progenitors that repopulate missing neurons and glia [1]. In the retinas of mice, frog and fish one population of cells that can be reprogrammed in response to injury is Müller glia [2]. In normal retina Müller glia have stem-like behaviors, dividing asymmetrically to produce progenitors of the rod photoreceptor lineage [3]. After photoreceptor or retinal neuron damage, Müller glia can dedifferentiate and produce progenitors that give rise to the major neural retinal cell types. The zebrafish retina has been used extensively as a model system to investigate the molecular mechanisms required for this process [4]–[6]. Major signal transduction pathways activated in reprogrammed Müller glia in zebrafish include EGF [7], Stat3 [8]–[10] and Wnt [11], [12]. Understanding how these signaling pathways promote glia reprogramming and neural regeneration is important for advancing treatments of central nervous system injury and disease.

In this study we present the characterization and molecular cloning of a zebrafish transgenic line *Tg(flk1:RFP)is18* that develops highly penetrant tumors in the retina and optic tract with features of retinoblastoma and fibrous glioma. The tumor phenotype is linked to a high copy number array of an RFP expressing reporter transgene in line *Tg(flk1:RFP)is18*. The transgene integrated in a long intergenic noncoding RNA gene *lincRNAis18*, which was previously identified in the zebrafish embryonic transcriptome as *Zv9_00007276 and Zv9_00007274* [13]. Isolation of a second targeted deletion allele of the *lincRNAis18* gene did not result in tumor formation, suggesting loss of function of the locus is not the initiating event that triggers tumor onset. Histological, cytological, and transcriptome analyses in pre-tumorous retina and tumor tissue reveal gene signatures of radial glia, neural progenitors, and injury induced activation of glia and astrocytes. The *Tg(flk1:RFP)is18* tumors are similar to the zebrafish model of optic pathway glioma driven by activated Sonic hedgehog signaling in neural progenitors [14]. Our analyses indicate the *Tg(flk1:RFP)is18* tumors originate from neural progenitors derived in part from an activated glial cell population that includes reprogrammed Müller glia.

## Results

### Isolation and molecular mapping of the optic pathway tumor line *Tg(flk1:RFP)is18*

**Figure 1:**
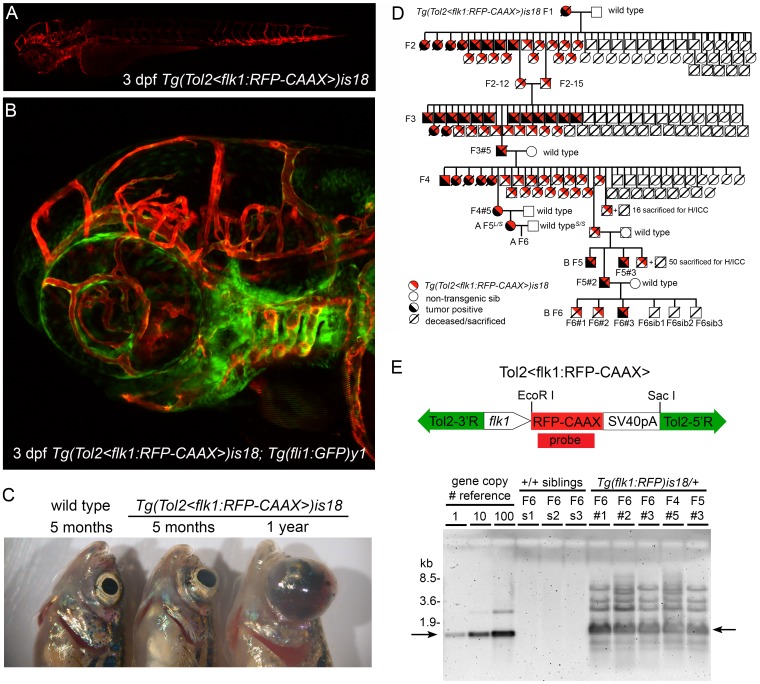
Genetic linkage of zebrafish ocular tumor model in transgenic line *Tg(flk1:RFP)is18*. (A) Endothelial-specific membrane targeted RFP expression in live 3 dpf zebrafish larva from transgenic line *Tg(flk1:RFP)is18*. (B) Endothelial-specific membrane targeted RFP expression and nuclear eGFP expression in live 3 dpf *Tg(flk1:RFP)is18; Tg(fli1:eGFP)y1* zebrafish larva. (C) Gross morphology of ocular tumors in heterozygous *is18/+* adults at 5 months and 1 year of age. (D) Pedigree of one family showing trans-generational linkage analysis of ocular tumor phenotype with inheritance of the *Tol2<flk1:RFP>* transgene in heterozygous *is18/+* individuals. Two families, A and B, were established from the F4 generation. Marked individuals were used in subsequent STR/STS linkage (L/S) and Southern blot analyses (#). (E) Diagram of endothelial-specific RFP reporter construct *pTol2<flk1:RFP-CAAX>* with positions of *EcoR*I and *Sac*I sites. Genomic Southern blot hybridized with RFP probe detects 1.3 kb *EcoR*I -*Sac*I fragment (arrows) of the *Tol2<flk1:RFP-CAAX>* transgene. The high copy-number *Tol2<flk1:RFP>* transgene array is transmitted specifically to RFP expressing, tumor positive *Tg(flk1:RFP)is18* progeny through the F6 generation.

We isolated a transgenic zebrafish line in which heterozygous adults develop neoplasia of the retina, optic nerve and optic tract. Transgenic line *Tg(Tol2<flk1:RFP-CAAX>)is18* (abbreviated as *Tg(flk1:RFP)is18)* was generated using a Tol2 transposon reporter construct that expresses membrane-targeted RFP-CAAX throughout vascular endothelial cells (Fig. 1A, B). Heterozygous *Tg(flk1:RFP)is18* adults developed large ocular tumors that first became evident at approximately 5 months of age (Fig. 1C) with a penetrance of >80%. Multigenerational genetic analysis demonstrated that the tumor phenotype was linked to inheritance of the *Tg(flk1:RFP)is18* transgene, as determined by RFP expression (Fig. 1D). In each generation non-transgenic siblings were healthy and showed no evidence of tumor formation. These results indicated that the tumor phenotype was due either to the location of the *Tg(flk1:RFP)is18* transgene in the genome or the presence of the transgene itself. Attempts to map the *Tg(flk1:RFP)is18* transgene integration site by standard ligation mediated PCR and inverse PCR methods suggested that during isolation of the line, multiple copies of the entire pTol2<flk1:RFP> construct, including the vector backbone, had integrated as a concatemer array. We confirmed by genomic Southern blot that the *Tg(flk1:RFP)is18* line contains an array with ∼100 copies of the *Tol2<flk1:RFP>* construct (Fig. 1E). Genomic DNA from *Tg(flk1:RFP)is18* individuals from 3 consecutive generations hybridized with an RFP-specific probe revealed multiple bands, including the expected 1.3 kb *EcoR*I-*Sca*I fragment from the *Tol2<flk1:RFP>* construct. In each generation non-transgenic siblings that lacked RFP expression in vascular tissue did not inherit the concatemer (Fig. 1E). Together these results provided strong evidence that the transgene in line *Tg(flk1:RFP)is18* is a stable, high-copy number concatemer that displays Mendelian transmission from one generation to the next.

**Figure 2:**
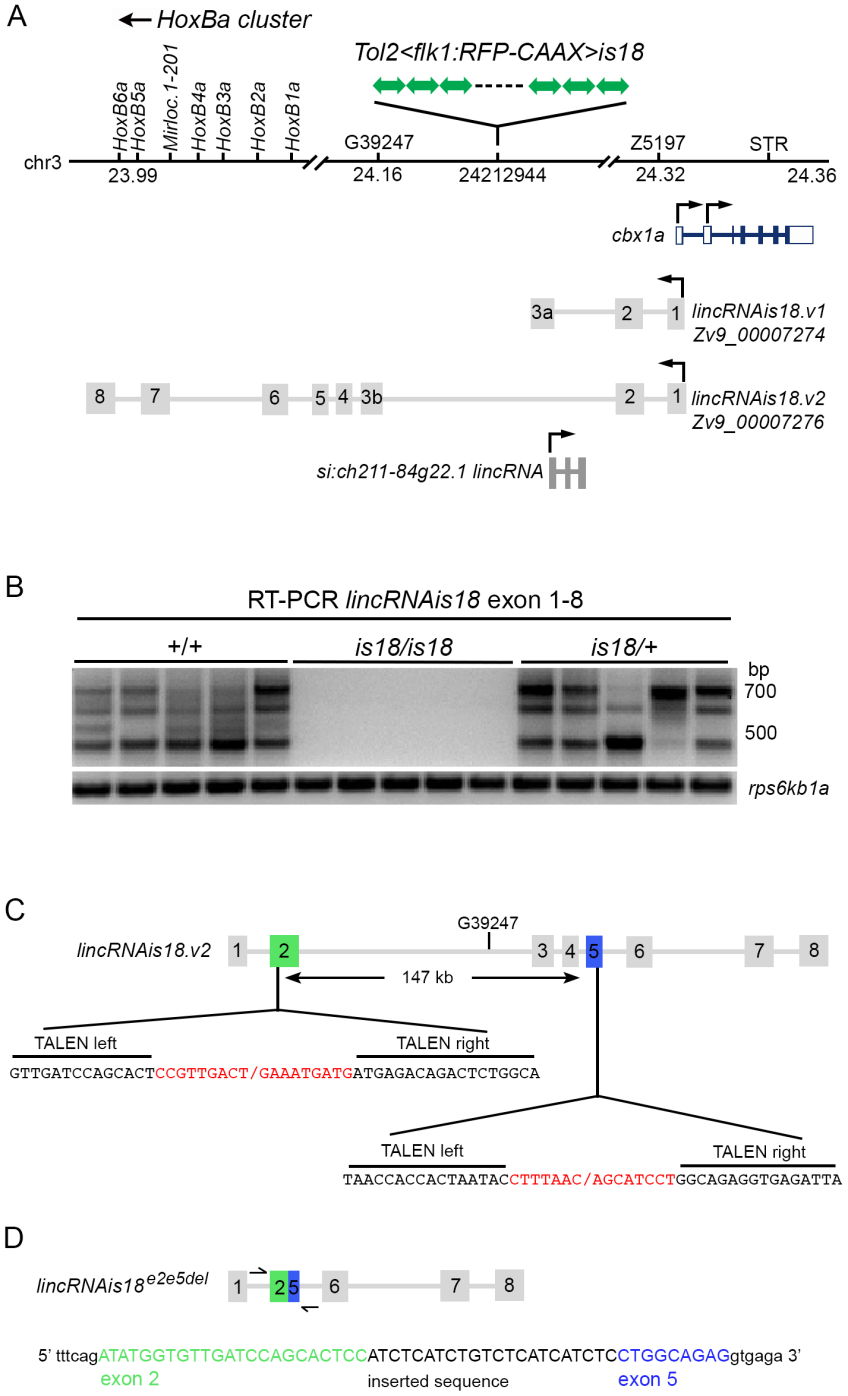
The *Tg(flk1:RFP)is18* transgene integration site on chromosome 3 disrupts expression of *lincRNAis18.v2*. (A) Integration site of the transgene array at 24.2 Mb on chromosome 3. The integration sits downstream of the *HoxBa* cluster and upstream relative to the heterochromatin binding family member *cbx1a*. *lincRNAis18* exon I overlaps with a 5′ regulatory region of the *cbx1a* gene. Positions of upstream and downstream microsatellite markers, G39247 and Z5197, and an STS/STR in *cbx1a*, are shown. (B) RT-PCR analyses with primers in exon 1 and exon 8 of *lincRNAis18.v2* showing expression of *lincRNAis1.v28* is disrupted in 6 dpf homozygous *Tg(flk1:RFP)is18* larvae. 5 individual larvae of each genotype are shown. The genotype of each larva was confirmed (Figure S6). Control, expression of ribosomal protein S6 kinase b, polypeptide 1, *rps6kb1*. (C) TALENs targeting exons 2 and 5 of *lincRNAis18*. (D) Predicted structure of the *lincRNAis18^e2e5del^* deletion allele. Sequence of amplicon spanning exon 2 – exon 5 junction from F1 *lincRNAis18^e2e5del^/+* adult genomic DNA.

To clone the genomic DNA flanking the *Tg(flk1:RFP)is18* concatemer integration site and map its location in the zebrafish genome, we designed a custom SureSelect Target Enrichment kit (Agilent Technologies) with single stranded biotinylated RNA probes that tile across the entire *pTol2<flk1:RFP>* vector (S1 A Figure; Table 1). Random sheared *Tg(flk1:RFP)is18* genomic DNA was hybridized to the biotinylated bait library in order to capture all of the concatemer transgene sequences. Since the genomic DNA was randomly sheared, we predicted a percentage of captured sequences would span the junction of the ends of the concatemer and the genomic DNA. Five independent genomic DNA samples isolated from muscle or tumor tissue, representing 3 *Tg(flk1:RFP)is18* individuals from 3 different generations, were captured and used to produce barcoded Illumina libraries for multiplex paired end sequencing (S2 Table). Sequences from the captured libraries were filtered to remove reads in which both ends mapped to the zebrafish genome or the *pTol2<flk1:RFP>* construct. The remaining paired end reads contained *Tol2<flk1:RFP>* transgene sequences at one end and zebrafish genomic sequences at the other. Four candidate integration sites that contained 9 or more reads from multiple samples (S3 Table) were chosen for further analysis. Three of the four sites mapped to regions with highly repetitive DNA, indicating non-specific capture of during hybridization. The fourth site had 80 reads that aligned to either side of position 24.212 Mb on chromosome 3 of the v9 zebrafish genome (S1 B Figure). This location maps in between the *HoxBa* cluster and the Hp1 heterochromatin binding protein family member *cbx1a* (Fig. 2A). The integration site was confirmed by sequencing of PCR amplification products that span the *Tg(flk1:RFP)is18* transgene-genomic DNA junction (S1 C, D Figure). An 8 bp duplication was present at the integration site, which indicated a simple *Tol2* transposon integration (S1 C Figure). However, additional molecular analyses confirmed that the high copy array detected by genomic southern (Fig. 1E) is also tightly linked to the integration site on chromosome 3.

**Table 1:** Linkage of *Tg(flk1:RFP)is18* transgene integration site to position 24.219 Mb Chromosome 3.

			Genotype		Haplotype Analysis#		Linkage Analysis$	
Primer pair	Marker*	Chr3 Position	is18/+ F5#3 female	WIK male	10 is18/+ siblings	10+/+ siblings	100 is18/+ offspring	100+/+ siblings
1	Z7419	23158018	L/M	M/S	6/10 L/M	6/10 M/M	57/100 L/M	NA
					4/10 L/S	4/10 M/S	42/100 L/S	NA
							1/100 M/M	NA
2	G39247	24163628	L/S	L/L	10/10 L/S	10/10 L/L	NA	NA
3	transgene -	24219944	+	-	10/10	0/10	100/100	0/100
	genomic DNA							
4	transgene	-	+	-	10/10	0/10	100/100	0/100
5	Z5197	24324461	L/S	S/S	10/10 L/S	10/10 S/S	100/100 L/S	NA
6	*cbx1a* STS	24360325	L/M	M/S	6/10 L/M	6/10 M/M	NA	NA
					4/10 L/S	4/10 M/S	NA	NA
7	Z7486	59983229	L/S	L/S	3/10 L/L	4/10 L/L	NA	NA
		59983229	L/S	L/S	4/10 L/S	2/10 L/S	NA	NA
		59983229	L/S	L/S	3/10 S/S	4/10 S/S	NA	NA

*Microsatellite STS markers Z7419, G39247, Z5197, Z7486. *cbx1a STS* is a Short Simple Repeat located in the 5th intron of the *cbx1a* gene. Genotype determined by size of PCR amplification products after gel electrophoresis. L, long; M, middle; S, short allele PCR products. *is18* transgenic genotype (+ or −) confirmed by PCR with primers that amplify across the genomic DNA-transgene junction (3) or amplify an internal fragment of the transgene concatemer (4).

# Genotype of *is18*/+ female and WIK male was determined. Segregation of STS markers in 10 is18/+ and 10+/+ siblings was used to determine the haplotype of the chromosome containing the is18 transgene integration. The *Tg(RFP)is18* chromosome haplotype in the region surrounding the integration site is Z7419-L, G39247-S, Z5197-L, *cbx1a* STS-L.

$ 100 *is18*/+ and 100+/+ siblings from a cross between a *Tg(flk1:RFP)is18/+* female and WIK wild type male were genotyped by PCR. 1/100 *Tg(flk1:RFP)is18/+* progeny was homozygous Z7419 M/M and heterozygous G39247 L/S. This indicates a single recombination event between markers Z7419 and G39247 and genetic map distance of 1 centimorgan.

Genomic Southern blot analysis of restriction fragment length polymorphism caused by the transgene integration confirmed the location of the *Tg(flk1:RFP)is18* concatemer on chromosome 3 (S2 and S3 Figures). Further confirmation of the location of the transgene concatemer was obtained by PCR linkage analysis with chromosome 3 sequence tagged site (STS) and short tandem repeat (STR) markers (S4 Figure). We identified a haplotype segregating on the transgene-containing chromosome 3 for 4 STS markers: Z7419, G39247, Z5197, and an STR in exon 5 of *cbx1a* (Table 1). PCR linkage analysis demonstrated that the *Tg(flk1:RFP)is18* chromosome 3 haplotype at marker Z5197 located ∼105 Kb from the integration site segregated with the transgene and tumor phenotype in 100 RFP expressing progeny from a tumor-bearing F5 adult *Tg(flk1:RFP)is18* female outcrossed to a WIK wild type male (Table 1). As expected, one recombinant was recovered between the transgene and marker Z7419 located ∼1.05 Mb 5′ to the integration site (Table 1). Together with the genomic Southern blot analyses, the linkage data provided strong evidence that the optic pathway tumor phenotype in line *Tg(flk1:RFP)is18* was due to the presence of a high copy number concatemer that had integrated at position 24.212 MB on chromosome 3.

### Zebrafish *Tg(flk1:RFP)is18* transgene integration in the *lincRNAis18* gene

We searched NCBI, Ensembl, and the recent zebrafish embryonic transcriptome study [13] for long intergenic noncoding RNAs (lincRNAs) that map to the region between the *HoxBa* cluster and *cbx1a* on chromosome 3 and identified two. One of the genes, *si:ch211-84g22.1* ENSDARG00000097724, was located ∼14 kb 3′ to the integration site (Fig. 2A). The second lincRNA was identified by a ∼930 bp transcript FDR202-P00026-DEPE-F_N10 FDR202 EH545544/Zv9_00007276 containing 8 exons that map to a genomic region spanning ∼350 kb (Fig. 2A). The 5′ region and first exon overlaps the *cbx1a* gene, and downstream exons 6, 7, and 8 map in the intergenic regions between genes in the *HoxBa* cluster (Fig. 2A). A shorter transcript of ∼480 bp (si:ch211-246i5.4 ENSDARG00000097621/FDR202-P00032-DEPE-R EH568666.1/Zv9_00007274) terminates in an alternative third exon. Neither transcript is predicted to encode a polypeptide longer than 73 amino acids (reading frame 3). We named the locus encoding these transcripts *lincRNAis18*. The shorter transcript is designated *lincRNAis18.v1*, the longer transcript is designated *lincRNAis18.v2* (Fig. 2A). The *Tg(flk1:RFP)is18* concatemer integration site is in the second intron of *lincRNAis18.v2* (Fig. 2A). Since the concatemer contains many copies of the SV40 bidirectional polyadenylation sequence, the transgene is predicted to cause premature transcriptional termination of *lincRNAis18.v2*. Together, the data showed that the optic pathway tumor phenotype was linked to the integration of the *Tg(flk1:RFP)is18* concatemer in the second intron of *lincRNAis18.v2.*

We performed a number of experiments to determine whether *lincRNAis18* is involved in formation of ocular tumors in *Tg(flk1:RFP)is18* heterozygotes. We examined the expression pattern of *lincRNAis18* by *in situ* hybridization and RT-PCR to determine whether it is expressed in the zebrafish retina. *in situ* hybridization on adult retina detected a low level of *lincRNAis18* expression in the inner nuclear layer and the ganglion cell layer (S5 A Figure), indicating it is not highly expressed in the retina. RT-PCR on an adult tissue panel revealed high levels of *lincRNAis18.v2* transcripts in the male and female germline and lower levels in muscle and retina (S5 B Figure). High levels of maternally supplied *lincRNAis18.v2* were observed in the developing embryo before the onset of zygotic transcription (S5 C Figure) then rapidly declined later stages. Multiple alternatively spliced forms of *lincRNAis18* were cloned from wild type adult retina, ovary and embryonic and larval stages (S5 Figure). Exons 1, 2 and 8 were consistently present in all isoforms, indicating a possible functional role of these exons. Although a low level of *lincRNAis18.v2* expression was detected in the retina, the specificity of expression in this tissue, and its absence from other adult tissues except muscle, suggested it may have a role in retina function.

To further examine the coding potential of *lincRNAis18*, we searched the recently published ribosome profiling datasets of expressed transcripts from 8 early developmental stages of zebrafish [15]. The datasets included 2–4 cell, 256 cell and 1000 cell embryos in which a high level of maternally supplied *lincRNAis18* is present. None of the ∼900 million 35 bp sequence reads fully aligned with the *lincRNAis18* sequence, providing further evidence that *lincRNAis18* represents a novel lincRNA that most likely is not translated into a functional protein. Database searches showed *lincRNAis18* has no significant homology with lincRNAs from other species. Despite the lack of homologous sequences in other vertebrate species, the high levels of *lincRNAis18* expression in the embryo suggests it could play an important role in early zebrafish development.

To determine whether the transgene integration in *Tg(flk1:RFP)is18* disrupts expression of *lincRNAis18.v2*, we examined *lincRNAis18.v2* expression by RT-PCR in homozygous *Tg(flk1:RFP)is18/Tg(flk1:RFP)is18* individuals. Progeny from an intercross of *Tg(flk1:RFP)is18/+* heterozygous adults were sorted into RFP-expressing and RFP-negative classes. Genomic DNA and total RNA were isolated from each individual for genotyping and RT-PCR. Expression of *lincRNAis18.v2* was undetectable in 5/5 homozygous *is18/is18* larvae (Fig. 2B). The genotype of each individual was confirmed by PCR (S6 Figure). These data demonstrate that integration of the *Tg(flk1:RFP)is18* transgene disrupts expression of the long form of *lincRNAis18*, *lincRNAis18.v2*.

To determine whether homozygosity results in additional phenotypes, all surviving progeny from the *is18/+* heterozygous intercross were raised and sacrificed at 4 weeks of age for PCR genotyping of genomic DNA. Some larvae began to develop edema by 7 dpf and as the larvae were aged many developed edema and did not survive. At 4 weeks of age, each of the 96 surviving RFP-expressing juveniles was sacrificed and genotyped. All were heterozygous for a wild type and a *Tg(flk1:RFP)is18* chromosome (data not shown), indicating that homozygous *Tg(flk1:RFP)is18/Tg(flk1:RFP)is18* individuals die from edema between 7 dpf and 4 weeks of age. These data indicate that homozygous *Tg(flk1:RFP)is18* larvae lack expression of *lincRNAis18.*v2 (Fig. 2B), due to premature transcription in the transgene, as predicted by the location of the transgene integration in intron 2. However, this does not rule out the possibility that the onset of edema and homozygous lethal phenotype might be caused by the presence of the *Tg(flk1:RFP)is18* transgene concatemer or its effect on nearby genes.

To test the hypothesis that disruption of expression of *lincRNAis18.v2* is homozygous lethal and involved in tumor formation, we isolated a second allele using TALEN genome editing to create a deletion in the *lincRNAis18* gene (Fig. 2C). TALENs targeting exons two and five (S7 Figure) were co-injected to create a deletion that removes 147 kb of genomic DNA and fuses exon two to exon five. Exons further upstream or downstream were not targeted in order to avoid affecting the genomic region surrounding the *cbx1a* and *HoxBa* cluster genes. We recovered 1 founder out of 27 that transmitted the deletion allele to F1 progeny. Sequencing of the exon 2 - exon 5 junction fragment and genotyping with STR markers confirmed the identity of F1 individuals carrying the deletion allele *lincRNAis18^e2e5del^* (Fig. 2D). To test whether the deletion allele was homozygous lethal, heterozygous *lincRNAis18^e2e5del^* F1 adults were intercrossed and the progeny were raised to adulthood. Genotyping identified homozygous F2 adults (S8 Figure), demonstrating that deletion of the genomic sequences between exons two and five of *lincRNAis18* did not result in lethality. By 8 months neither heterozygous nor homozygous *lincRNAis18^e2e5del^* adults developed ocular tumors. Together, these data suggest that tumor onset in *Tg(flk1:RFP)is18* individuals is most likely not due to a loss of function of the *lincRNAis18* gene, or disruption of expression of *lincRNAis18.v2*. However, since the deletion allele that was generated is predicted to produce a transcript containing exons 1, 2/5, 6, 7 and 8, it does not represent a null allele, and may retain some function that prevents homozygous lethality and tumor onset. Alternatively, because the *Tg(flk1:RFP)is18* transgene mutation segregates as a dominant allele, it might create a dominant effect on the expression of other *lincRNAis18* transcript isoforms. It is also possible that the presence of the high copy number transgene in *Tg(flk1:RFP)is18* adults induces ocular tumor formation through an oncogenic mechanism that induces overexpression of nearby genes. Transcriptome analyses presented below do not support the latter mechanism.

### Histopathology of the zebrafish *Tg(flk1:RFP)is18* optic pathway tumors reveals features of retinoblastoma and fibrous glioma

**Figure 3:**
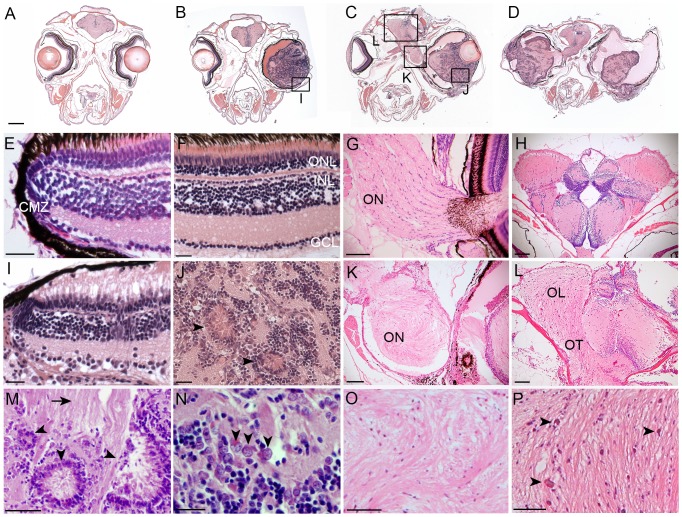
Histological analysis of *Tg(flk1:RFP)is18* tumors reveals similarities with retinoblastoma and glioma. Coronal sections through heads of wild type (A, E–H) and *Tg(flk1:RFP)is18/+* adults (B–D, I–L). (B) 5-month-old *Tg(flk1:RFP)is18/+* adult with retinal tumor filling the vitreous. 1-year-old *Tg(flk1:RFP)is18/+* adults with unilateral (C) or bilateral tumors (D). Tumor cells extend through the optic pathway to the tectum. (E,F) Ciliary marginal zone and mature retina in wild type. (G) Fibrillar ribbon-like structure of a wild type optic nerve exiting the eye. (H) Section through forebrain shows the sacus dorsalis and left and right lobes of the anterior region of the optic tectum. (I) Intact ciliary marginal zone in the tumorous retina from *Tg(flk1:RFP)is18/+* adult shown in B. Degeneration of the retinal pigment epithelium and photoreceptor outer segments is evident. Disorganization of the retinal layers adjacent to the ciliary marginal zone is present. Streaking across the inner plexiform layer appears similar to reactive Müller glia. (J) Advanced tumors contain rosettes and blood vessels extending throughout the tumor tissue. (K) Disorganization and dysplasia in the optic nerve of the *Tg(flk1:RFP)is18/+* adult in C. (L) Brain from the adult in panel D showing dysplasia of the optic tract with infiltration and disruption of normal brain structures. (M) Dysplastic areas of advanced tumor with rosettes of various sizes (arrowheads) and extensive glial fibrillar proliferation (arrow). (N) “Salt and pepper” chromatin dispersion pattern (arrowheads) consistent with a neuroectodermal tumor cell. (O) Disorganization of the optic nerve with absence of organized fibrils. (P) Expansion of the optic lobe with possible areas of necrosis (arrowheads). CMZ, ciliary marginal zone; GCL, ganglion cell layer; INL, inner nuclear layer; OL, optic lobe; ON, optic nerve; ONL, outer nuclear layer; OT, optic tract. Scale bars A, B, C, D 500 µm; E, F, G, I, J, N 20 µm; H, K, L 100 µm; M, O, P 50 µm.

Histopathology of the tumor positive fish revealed large intraocular masses that filled the vitreal space and displaced the lens (Fig. 3B–D). By one year of age affected individuals frequently developed tumors in both eyes (Fig. 3D). Advanced retinal tumors were not characterized by necrosis or high mitotic activity. The tumors were composed of neuroepithelial-like cells with areas of dysplastic tissue forming rosettes of various sizes (Fig. 3J, M arrowheads). Blood vessels were present throughout the tumors, indicating neovascularization in the advanced tumors. There were extensive glial fibrillar structures within the tumors (Fig. 3J, M arrow) and the optic nerve and tract (Fig. 3K, L). In advanced tumors the optic nerves and tract were completely replaced by neoplastic tissue, and the architecture of the brain lobes was highly disrupted (Fig. 3C, D, K, L). Cells having a “salt and pepper” chromatin dispersion pattern were present, again consistent with neuro-ectodermal tumor cell cytomorphology (Fig. 3N, arrowheads). Areas of necrosis were present throughout the optic lobe (Fig. 3P arrows). Examination of tumors in younger adults revealed relatively normal organization of the retina at the ciliary marginal zone. In a 5-month old adult with a large ocular mass (Fig. 3B), the neural retinal layers were observed to be intact at the ciliary marginal zone (Fig. 3I). These initial examinations indicated the ocular masses likely originated from cells located in the differentiated tissue of the neural retina, not from the retinal stem cell population that resides at the ciliary marginal zone. Overall, the retinal tumors appeared similar to fibrous glioma with features present in human retinoblastoma.

### Evidence for involvement of glia in *Tg(flk1:RFP)is18* tumor proliferation

**Figure 4:**
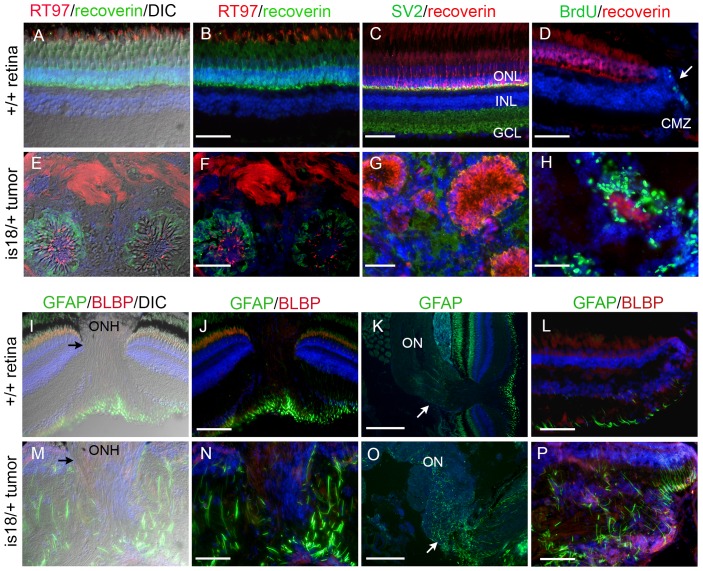
Characterization of *lincRNAis18* tumors indicates a glial cell origin. Immunolabeling and in situ hybridization of cryosections from wild type retina (A–D, I, J, M–P) and advanced *lincRNAis18* tumors (E–H, K, L, Q–T). Cells in rosettes in the tumors label with neurofilament marker RT-97 (red) and photoreceptor marker recoverin (green) (E, F). The synaptic vesicle marker SV2 (green), which is enriched in the retinal plexiform layers (C), was distributed throughout the fibrous tumor mass (G). (D) BrdU (green) incorporated into proliferating progenitor cells at the ciliary marginal zone of the wild type retina (arrow). (H) Intense labeling of BrdU incorporation was detected in cells forming rosettes and throughout the tumor mass. In wild type the glial marker GFAP is most evident in the Müller glia end feet that sit in the retinal ganglion cell layer (I–L). GFAP expression is absent in the oligodendrocytes and astrocytes of the optic nerve (I, K arrow). In contrast, GFAP was readily detected in long streaks throughout the tumor tissue (M, N, P), and was expressed by cells in the mutant optic nerve (O, arrow). BLBP is present at the ciliary marginal zone of wild type retina (L) and appeared present throughout the tumor mass (P). A, E, I, M, Differential interference contrast (DIC) overlay on immunofluorescence labeling images. CMZ, ciliary marginal zone; GCL, ganglion cell layer; INL, inner nuclear layer; ON, optic nerve; ONH, optic nerve head; ONL, outer nuclear layer. All scale bars represent 50 µm, except in panels K and O scale bars represent 100 µm.

To gain further insight into the identity of the *Tg(flk1:RFP)is18* retinal tumors we examined the expression of retinal neural markers in cryosections of tumor tissue. The calcium binding protein Recoverin is normally expressed in all photoreceptors in the retina (Fig. 4A), while the antibody RT97, which broadly recognizes neuronal intermediate filament proteins, also labels photoreceptor outer segments (Fig. 4A). In tumors Recoverin was detected in the cell bodies and RT97 labeled structures projecting into the center of the rosettes (Fig. 4E–H). RT97 also strongly labeled the fibrous stroma of the tumor (Fig. 4E–F), indicating the presence of neuronal processes in this matrix. The synaptic vesicle marker SV2 was also detected throughout the fibrous stroma (G). These analyses were consistent with the histopathology suggesting the tumors likely originate from the neural retina.

To examine the proliferative cell populations in the *Tg(flk1:RFP)is18* retinal tumors, we labeled adult fish with BrdU for 2 hours, followed by a 4 hour recovery period. In wild type retina BrdU incorporation was detected at the ciliary marginal zone where progenitor cells reside (Fig. 4D, arrow). Very rarely BrdU was detected in single cells in more central retina, consistent with the slow cycling of the Müller glia-rod photoreceptor lineage. We examined BrdU incorporation in advanced tumors and found robust labeling throughout the tumor mass with some overlap with rosettes (Fig. 4H). BrdU did not consistently co-label cells expressing a specific retinal neural cell type marker, making it difficult to conclusively determine the identity of proliferating cells in very advanced tumors. Moreover, histopathology did not indicate high mitotic activity in the advanced tumors (Fig. 3). This suggests the possibility that the large number of cells that incorporated BrdU was not due to DNA synthesis during S phase but was the result of the activity of global DNA repair processes.

Numerous studies have demonstrated that the Müller glia can be activated in response to retinal injury and reprogrammed to produce progenitors that repopulate all retinal neural cell types [16]. To determine if the *Tg(flk1:RFP)is18* tumors showed evidence of reprogrammed Müller glia-derived progenitors, we examined the expression of Müller glia and activated Müller glia markers by immunolocalization. The glial specific marker GFAP was detected in the Müller glia end feet located in the ganglion cell layer in wild type retina (Fig. 4I–L). Very little GFAP expression was detected in the glia of the wild type optic nerve (Fig. 4K, arrow). In the *Tg(flk1:RFP)is18* retinal tumors intense labeling of GFAP was detected extending throughout the tumor mass (Fig. 4M, N), indicating a glial cell type. Increased expression was also evident in the dysplastic optic nerve at the lamina cribrosa of an eye with an advanced tumor (Fig. 4O), indicating expansion of the astroglia of the optic nerve. To visualize activated Müller glia, we examined immunolocalization of Brain Lipid Binding Protein/fatty acid binding protein (BLBP/fabp). In wild type retinas BLBP is expressed in progenitor cells at the ciliary marginal zone (Fig. 4L) but is absent from Müller glia. In the *Tg(flk1:RFP)is18* retinal tumors an increase in BLBP was detected throughout the tumor and did not appear to co-localize with GFAP positive Müller glia cells (Fig. 4L, P). This suggested BLPB was labeling a population of cells distinct from mature Müller glia. Together these results indicate *Tg(flk1:RFP)is18* tumors might be the result of abnormal levels of glial proliferation, with features similar to activated Müller glia and the neural progenitors derived from them.

### Differential gene expression analysis of *Tg(flk1:RFP)is18* retinal tumor progression

To identify the molecular signatures that correlate with onset and tumorigenesis of *Tg(flk1:RFP)is18* tumors we performed differential gene expression analysis by RNA-Seq. Libraries were prepared from 6 month old age-matched wild type retina (Wild Type), pretumor retina from heterozygous *Tg(flk1:RFP)is18* adults (Pretumor), and advanced tumor tissue from heterozygous *Tg(flk1:RFP)is18* adults (Tumor) (S6 Table). The data was mapped to the zebrafish v9 genome, gene model assembly version 71 (S7 Table). The gene models included 1133 recently identified lincRNAs expressed during early zebrafish development [13]. Genes with FPKM (Fragments Per Kilobase per Million sequenced reads) value of > = 1 in Wild Type retina were examined for significant changes in expression level in Pretumor and Tumor samples (S7 Table). GO term analysis (Table 2) indicated translational activity was elevated in Pretumor and Tumor tissue. In Pretumor and Tumor tissue cellular respiration was decreased; photoreception and ion transport processes were also decreased in tumor. Consistent with the GO term analysis, the gene set that showed the greatest down-regulation between Wild Type and Tumor was enriched for genes that function in photoreceptor maintenance and photoreception (S8 Table), indicating that in the *Tg(flk1:RFP)is18* tumors, normal photoreception and synaptic transmission are disrupted. As expected for proliferating tumor, mitosis and cell division processes were increased in *Tg(flk1:RFP)is18* tumors. 252 of the 1133 lincRNAs identified in the zebrafish embryonic transcriptome were expressed in wild type retina (S7 Table). 11 of the 252 lincRNAs were significantly increased or decreased in expression level in Pretumor and/or Tumor tissue (S9 Table).

**Table 2:** Categories of biological processes altered in *Tg(flk:RFP)is18/+* Pre-tumor and Tumor tissues.

DGE Group*	GO Process	p-value
**Increased in Pretumor**	translation	4.50e-13
	ribonucleoprotein complex biogenesis	9.59e-8
	regulation of translational initiation	5.62e-5
	ribosome biogenesis	9.28e-5
**Decreased in Pretumor**	cellular respiration	9.38e-7
	energy derivation by oxidation of organic compounds	4.53e-5
	ATP metabolic process	9.01e-5
**Increased in Tumor**	ribonucleoprotein complex biogenesis	1.99e-13
	translation	3.86e-12
	mitotic cell cycle	9.48e-9
	cell division	5.02e-7
**Decreased in Tumor**	sensory perception of light stimulus	2.00e-4
	cellular respiration	3.11e-3
	ion transport	3.67e-3

*DGE Group: Differential Gene Expression Group. Genes were grouped according to direction of change in expression level in Pretumor and Tumor tissue.

We examined the expression levels of genes that map in the region of the *Tg(flk1:RFP)is18* transgene integration in order to determine if the array influenced expression of nearby genes. Only 5 reads in the wild type retina transcriptome mapped to *lincRNAis18*, indicating the majority of *lincRNAis18* mRNA was lost during isolation of polyadenylated RNA for RNA-Seq library construction. We confirmed this by RT-PCR using the input RNA for library construction and samples of the resulting libraries. The presence of *lincRNAis18* mRNA was detected by RT-PCR in the total RNA input sample, but was only detectable in the wild type RNA-Seq library after nested RT-PCR (S9 Figure). The lincRNA *si:ch211-84g22.1*, located in intron 2 of *lincRNAis18* and positioned 14 kb 3′ to the *Tg(flk1:RFP)is18* transgene integration site, was only represented by 4 reads in wild type retina. Expression levels of *si:ch211-84g22.1* in *Tg(flk1:RFP)is18* Pre-Tumor and Tumor tissue were increased (98 and 132 reads total), but did not significantly change between the two samples. These results raise the possibility that the dominant nature of the *Tg(flk1:RFP)is18* transgene to induce tumors could be the result of activation of expression of the nearby lincRNA *si:ch211-84g22.1.* Other genes located in the region of the *Tg(flk1:RFP)is18* transgene integration were examined for altered expression. No significant change in expression level was detected for genes that map within 1 Mb upstream or downstream of the transgene integration, including the *HoxBa* cluster and *cbx1a* genes. Overall, the analyses indicate the *Tg(flk1:RFP)is18* transgene integration is responsible for inducing tumor formation, but the mechanism does not involve altering the expression level of genes in cis other than the lincRNA *si:ch211-84g22.1*.

#### Human Mutation-Driver and Glioma Marker Genes

**Figure 5:**
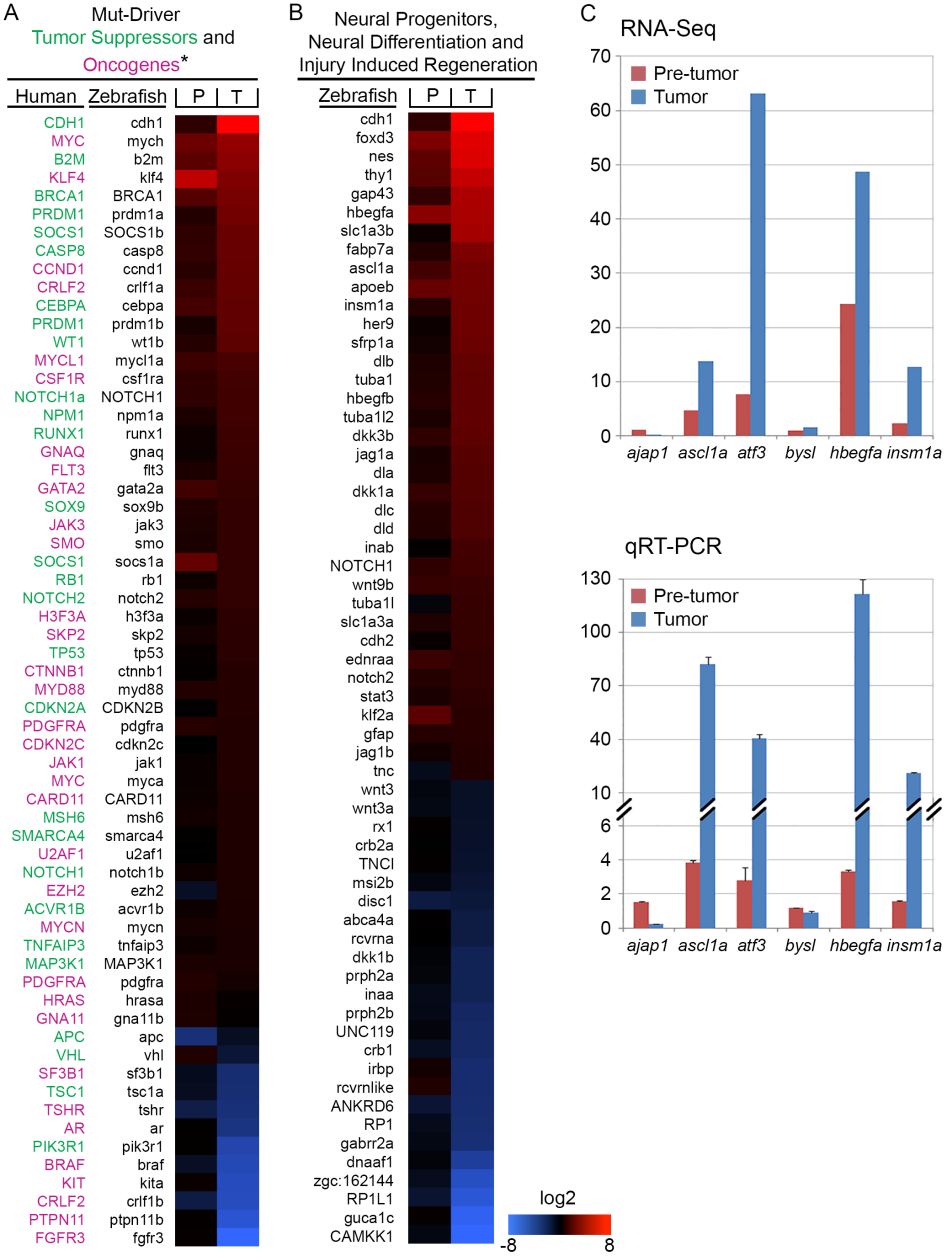
Differential gene expression analysis of retina RNA-Seq from wild type, pre-tumor, and retinal tumor tissues. A, B Heat map representations of differentially regulated transcripts associated with tumor initiation and progression. (A) Differential gene expression of 62 Mutation-Driver genes. Tumor suppressors are in green, oncogenes are in red. (B) Differential gene expression of genes required for photoreceptor and neural function, neural progenitor proliferation, and injury induced regeneration. Scale represents log2 fold change in gene expression. (C) Comparison of absolute fold change in gene expression levels for *ajap1*, *ascl1a*, *atf3*, *bysl*, *hbegfa*, and *insm1a* measured by RNA-Seq (upper panel) and qRT-PCR (lower panel). *Tg(flk1:RFP)is18/+* Pretumor (red bars) and *Tg(flk1:RFP)is18/+* Tumor (blue bars).

We examined the *Tg(flk1:RFP)is18* tumor differentially expressed gene set for changes in tumor suppressors and oncogenes that identify disrupted signal transduction pathways in cancer. Although the ocular tumors showed features of retinoblastoma tumors, the expression level of the *rb1* tumor suppressor was unchanged in pretumor and elevated ∼3 fold in tumor tissue (S7 Table), indicating the tumors do not arise due to deletion of the *rb1* locus. 62 of the 138 designated Mutation-Driver human cancer genes [17] showed at least a 2-fold change in expression (Fig. 5A). In the Wnt signaling pathway, two direct molecular targets of ß-catenin transcriptional activation, the *myc* oncogene [18] and cell cycle regulator *cyclinD1* [19], showed significant increases in expression in *Tg(flk1:RFP)is18* tumor tissue. Two tumor suppressors in the PI3K-mTOR pathway altered in the majority of human gliomas [20]–[23], PI3K regulatory subunit *pi3kr1* and the *tsc1* repressor of mTOR, were decreased in expression level in the *Tg(flk1:RFP)is18* tumors (Fig. 5A and S7 Table). The tumors showed a significant increase in expression of glioma diagnostic markers [24], [25] [22] *vimentin* (*vim*), *S100ß* (*s100ß*) and the matrix metalloprotease *mmp9* (Table 3). The zebrafish homolog of the putative tumor suppressor *ajap1* [26] was also significantly decreased in expression in *Tg(flk1:RFP)is18* tumor tissue (Table 3). qRT-PCR confirmed *ajap1* decreased in expression 4-fold (Fig. 5C). These data are consistent with the histological and immunohistochemical data that suggests *Tg(flk1:RFP)is18* retinal tumors express a glial gene signature.

**Table 3:** Differential Expression of Neural Progenitor, Müller glia Regeneration, and Glioma Genes in *Tg(flk1:RFP)is18/+* tumor progression.

			Fold Change	
Gene	Gene Name	Zebrafish Gene ID and Symbol	WT –>Pretumor	WT –>Tumor
**Neuroglial Progenitors**				
*atf3*	activating transcription factor 3	ENSDARG00000007823_atf3	7.7	63.1
*foxd3*	forkhead box D3 transcriptional activator	ENSDARG00000021032_foxd3	17.7	177.7
*gap43*	growth associated protein 43, nerve growth factor	ENSDARG00000015775_gap43	3.1	52.8
*nestin*	neural stem cell intermediate filament protein	ENSDARG00000088805_nes	8.7	162.1
*slc1a3a*	glutamate transporter GLAST/SLC1A3	ENSDARG00000026218_slc1a3a	2.1	3.4
*slc1a3b*	glutamate transporter GLAST/SLC1A3	ENSDARG00000043148_slc1a3b	1.3	43.6
*sox4a*	SRY-box-related 4a transcriptional activator	ENSDARG00000004588_sox4a	2	5.4
*sox4b*	SRY-box-related 4b transcriptional activator	ENSDARG00000043235_sox4b	1.6	6.1
*sox11a*	SRY-box-related 11a transcriptional activator	ENSDARG00000077811_sox11a	1.2	4.4
*sox11b*	SRY-box-related 11b transcriptional activator	ENSDARG00000095743_sox11b	2.3	18.5
*sox21b*	SRY-box-related 21b transcriptional activator	ENSDARG00000008540_sox21b	13	469
**Muller glia** *				
*apoeb*	apolipoprotein Eb	ENSDARG00000040295_apoeb	10.7	13.7
*blbp*	brain lipid binding protein	ENSDARG00000007697_fabp7a	2.2	17.7
*cdh2*	neuronal cadherin 2	ENSDARG00000018693_cdh2	1.2	3.4
*hes1*	hairy/enhancer of split 1 transcriptional repressor	ENSDARG00000056438_her9	1.3	12.6
*gfap*	glial fibrillary acid protein	ENSDARG00000025301_gfap	2.3	2.5
*rlbp1a*	retinaldehyde binding protein 1	ENSDARG00000012504_rlbp1a	1.9	1.3
*rlbp1b*	retinaldehyde binding protein 1	ENSDARG00000045808_rlbp1b	2.4	2.1
**Müller glia regeneration** #				
*ascl1a*	achaete-scute like 1a transcriptional activator	ENSDARG00000038386_ascl1a	4.6	13.8
*dkk1b*	dickkopf 1, wnt antagonist	ENSDARG00000045219_dkk1b	0.9	0.2
*hbegfa*	heparin binding epidermal-like growth factor	ENSDARG00000075121_hbegfa	24.4	48.8
*hbegfb*	heparin binding epidermal-like growth factor	ENSDARG00000031246_hbegfb	2.4	9.4
*insm1a*	insulinoma 1a transcriptional repressor	ENSDARG00000091756_insm1a	2.3	12.7
*stat3*	signal transduction and activator of transcription 3	ENSDARG00000022712_stat3	1.9	2.9
**Glioma genes** $				
*ajap1*	Adherens Junction Associated Protein 1	ENSDARG00000038655_ajap1	1	0.2
*mmp9*	matrix metalloprotease 9	ENSDARG00000042816_mmp9	2.0	7.4
*S100ß*	neural calcium binding protein	ENSDARG00000057598_s100b	1.6	5.0
*vim*	vimentin	ENSDARG00000010008_vim	2.8	9.6

*Raymond et al., 2006.

# Kassen el al., 2009; Nelson et al., 2012; Ramachandran et al., 2011; Ramachandran et al., 2012; Wan et al., 2012.

$ Zhao et al., 2010; Lin et al., 2012.

#### Glial activation and Neuronal Progenitors

Two populations of glia cells in the retina may contribute progenitor cells that give rise to the *Tg(flk1:RFP)is18* tumors. The Müller glia reside within the neural retina and in the mature retina function as late retinal progenitors that produce the rod photoreceptor lineage. The nerve fiber layer contains astrocytes that migrate into the retina from the optic nerve during embryogenesis. We examined the transcriptome data for changes in gene expression of markers for glia, glial progenitors, reactive astrocytes and activated Müller glia. Glutamate transporter GLAST/SLC1A3 has been identified as a marker of radial glia, glial progenitor cells, and glial progenitor-derived astrocytes [27]. Zebrafish *slc1a3a* and *-b* were both significantly increased in expression in the *Tg(flk1:RFP)is18* tumors (Table 3). Activating transcription factor 3 (*ATF3*) is a member of the cAMP-response element binding protein family of transcriptional activators that bind the CRE (cAMP response element) enhancer and are activated in response to a rise in cytosolic cAMP levels. *atf3* expression increases in the retinal ganglion and nerve fiber layers of the retina and the optic nerve after injury to the optic nerve in adult zebrafish [28]. In the *Tg(flk1:RFP)is18* transcriptome *atf3* expression levels increased significantly in Pretumor and Tumor tissue (Table 3). In contrast, *bystin* (*bysl)*, whose expression increases substantially after optic nerve injury and marks reactive astrocytes [29], remained unchanged (S7 Table). Changes in expression levels of *atf3* and *bysl* were confirmed by qRT-PCR (Fig. 5C). These data indicate reactive gliosis was not solely responsible for the increased expression of glial progenitor markers in the *Tg(flk1:RFP)is18* ocular tumors.

Many of the genes required for proliferation of neuroglial progenitor cells were increased in expression level in the *Tg(flk1:RFP)is18* tumor tissue. This included nerve growth associated factor *gap43*, neural stem cell intermediate filament protein *nestin*, and neural cadherin 2 *cdh2* (Table 3). Transcription factors required in neural stem cells (*foxd3*, *sox21b*, *sox4a*, *sox4b*, *sox11a* and *sox11b*) were also increased in *Tg(flk1:RFP)is18* Tumor tissue (Fig. 5B; Table 2). In response to photoreceptor degeneration after retinal injury, the Müller glia become activated to reenter the cell cycle and produce retinal progenitors. The gene expression pattern that defines proliferating Müller glia (*apoeb*, *blbp*, *cdh2*, *hes1*, *rlbp1a* and *–b*) also showed increased expression in the *Tg(flk1:RFP)is18* tumors. Reprogramming of Müller glia is mediated by activation of multiple signal transduction pathways [4]. The JAK/Stat cytokine signal transduction pathway transcription factor *stat3*, which is required for proliferation after light dependent retinal injury and regeneration [9], [10], was elevated nearly 3-fold in the tumor (Table 3). *Wnt*-ß-catenin signal transduction is activated in Müller glia derived progenitors via a network involving heparin binding epidermal growth factor *hbegf*, the transcriptional activator *ascl1a*, and the transcriptional repressor *insm1a* [7], [12]. Each of these genes was significantly increased in the *Tg(flk1:RFP)is18* Tumor, while the *wnt* antagonist *dkk1b* was significantly decreased in expression (Table 3). We confirmed the differential gene expression of *hbegfa*, *ascl1a*, and *insm1a* by qRT-PCR (Fig. 5C). Together, these data are consistent with the hypothesis that *Tg(flk1:RFP)is18* retinal tumors might arise from neural progenitors derived from Müller glia and/or astroglia.

## Discussion

In this report we describe the isolation and characterization of a zebrafish optic pathway tumor line that is linked to integration of a Tol2<flk1:RFP-CAAX> transgene concatemer in line *Tg(flk1:RFP)is18.* By 1 year, greater than 80% of heterozygous *Tg(flk1:RFP)is18* adults develop tumors in the retina, optic nerve and optic tract with features of retinoblastoma and fibrous glioma. Histological, immunohistochemical, and transcriptomic analyses of *Tg(flk1:RFP)is18* ocular tumors are consistent with the tumor originating in part from a glial cell population in the retina that includes the Müller glia. Astrocytes residing within the optic nerve fiber layer may also contribute to the retinal tumor cell progenitor population. The dominant pattern of inheritance indicates the presence of the transgene results in an oncogenic mechanism that induces tumor onset. The *Tg(flk1:RFP)is18* transgene integration is homozygous lethal, and animals die between 1 and 4 weeks of age. The transgene disrupts expression of the lincRNA gene, *lincRNAis18* (Zv9_00007276) but elevates expression of the opposite strand *lincRNA si:ch211-84g22.1* ∼25 fold. A second deletion allele generated using TALEN genome editing that removes exons 2–5 of *lincRNAis18* and the entire lincRNA *si:ch211-84g22.1* locus did not result in a tumor or lethal phenotype. This suggests that in line *Tg(flk1:RFP)is18* the presence of the Tol2<flk1:RFP-CAAX> array is responsible tumor formation and lethality, however, the mechanism underlying tumor onset is not known.

Our study provides several pieces of evidence that the *Tg(flk1:RFP)is18* retinal tumors likely originate from glial-derived neural progenitors in the retina. The *Tg(flk1:RFP)is18* phenotype is very similar to the previously reported zebrafish optic pathway glioma model in which activation of Sonic hedgehog signaling in neural progenitors induces tumors [14]. Radial glia in the CNS are the source of neural stem cells and glial progenitors during development and in adult neurogenesis [30]. The dramatic increase in expression of the radial glial marker *GLAST/slc1a3b* [27] and the neural stem cell markers *foxd3*, *nestin*, *sox4*, *sox11*, and *sox21* in *Tg(flk1:RFP)is18* tumor tissue supports a neuroglial progenitor population. The CREB transcriptional activator ATF3 and the reactive astrocyte marker Bystin are associated with reactive gliosis and upregulated in the optic nerve in response to injury [28], [29]. While *atf3* was dramatically increased in expression in the *Tg(flk1:RFP)is18* retinal tumors, zebrafish *bystin-like* (*bysl*) showed no change in expression level. Consequently, the activation of glia in the *Tg(flk1:RFP)is18* model is not merely a result of reactive gliosis due to insult or injury in the retina. ATF3 is a member of the cAMP-response element binding protein family of transcriptional activators that bind the CRE (cAMP response element) enhancer and are activated in response to a rise in cAMP levels. In fetal brain synergism between the cytokine LIF and BMP signal transduction pathways is mediated by CREB, which acts as a bridge between STAT3 and smad1 to promote astrocyte formation from neural progenitors [31]. Consistent with increase in CREB family member ATF3, both zebrafish *smad1* and *stat3* expression levels were elevated in *Tg(flk1:RFP)is18* tumors, indicating a role for cAMP signal transduction in tumor growth.

Activation of multiple signal transduction pathways required for inducing Müller glia proliferation and progenitor production was observed in the *Tg(flk1:RFP)is18* ocular tumors. Müller glia markers, such as apolipoprotein Eb, *apoeb*, brain lipid binding protein, *blbp*, and hairy/enhancer of split 1 transcriptional repressor, *hes1* [32], were significantly increased in the tumors. The JAK/Stat signal transduction and transcriptional activator *stat3*, which is required to stimulate Müller glia proliferation in the injured zebrafish retina [9], [10], was elevated as well in tumor tissue. The *insm1a*, *ascl1a/dkk1b*, and *hbegf* pathways that active *wnt* signaling to induce Müller glia proliferation [7], [11], [12] were also significantly altered. Together these data support the hypothesis that the Müller glia in the *Tg(flk1:RFP)is18* retinas dedifferentiate and produce transformed neuroglial progenitors. Retinal tumor progenitors might also arise from astrocytes located in the nerve fiber layer. Overall, the transcriptome data, histological analyses, and immunohistochemical labeling of tumor tissue provides significant support for the conclusion that transformed glia give rise to the *Tg(flk1:RFP)is18* tumors. Identifying the mechanism that initiates glial progenitor proliferation in the *Tg(flk1:RFP)is18* retina will require additional studies. The zebrafish *Tg(flk1:RFP)is18* line presents a highly penetrant and consistent retinal tumor model that will be useful for investigating the mechanisms driving glia activation and reprogramming in the vertebrate central nervous system.

## Materials and Methods

### Zebrafish husbandry and genetics

Zebrafish were reared in an Aquatic Habitat system (Aquatic Ecosystems, Inc., Apopka, FL). Fish were maintained on a 14-hr light/dark cycle at 27°C. Transgenic lines were established in a WIK wild type strain obtained from the Zebrafish International Research Center (http://zebrafish.org/zirc/home/guide.php). For in situ hybridization experiments, embryos were collected and maintained at 28.5°C in fish water (60.5 mg ocean salts/l) containing 0.003% 1-phenyl-2-thiourea (PTU) until harvesting. Embryos were staged according to published guidelines [33]. All experimental protocols were approved by the Iowa State University Institutional Animal Care and Use Committee (Log # 11-06-6252-I) and are in compliance with American Veterinary Medical Association and the National Institutes of Health guidelines for the humane use of laboratory animals in research. Adult fish were anesthetized and euthanized in MS-222 Tricaine Methanesulfonate prior to sacrifice and tissue dissection for histopathology and immunolabeling.

### Animal Care and Humane Endpoint Establishment

Transgenic fish predisposed to tumor formation were raised side by side with non-transgenic siblings. Heterozygous and homozygous transgenic fish and sibling fish were monitored daily during routine feeding for viability and morbidity, and monitored bi-weekly for gross presentation of ocular tumors. 50% of each generation of transgenic fish developed pericardial edema. All homozygous transgenic fish presented with pericardial edema beginning at 2–7 dpf and developing through 4 weeks of age. Fish presenting with edema were sacrificed before swimming and feeding behavior were adversely affected. Juvenile and adult fish were anesthetized and euthanized in MS-222 Tricaine Methanesulfonate according to experimental protocols approved by the Iowa State University Institutional Animal Care and Use Committee (Log # 11-06-6252-I) in compliance with the American Veterinary Medical Association and the National Institutes of Health guidelines for the humane use of laboratory animals in research.

NIH/Office of Animal Care and Use/Animal Research Advisory committee (ARAC) Guidelines for endpoint in neoplasia studies (oacu.od.nih.gov/ARAC/Guidelines for Endpoints in Animal Study Proposals) were used to establish a humane endpoint in the zebrafish heterozygous *Tg(flk1:RFP)is18* glioma tumor model. Adult fish were monitored bi-weekly for general appearance and assessed for size and length relative to non-transgenic siblings. Fish were sacrificed before tumor burden reached 3 mm in size/25 mg in weight, constituting less than 10% of the total body weight of an adult fish (300–500 mg), as outlined for mouse and rat studies [34]. No adverse affect on growth rate, feeding behavior or fertility was detected in *Tg(flk1:RFP)is18* fish with a tumor burden less than 3 mm/10% of body weight. For transcriptome studies age matched 6 month old fish with tumor size ranging from undetectable to 2 mm in size were anesthetized and euthanized in MS-222 Tricaine Methanesulfonate. Retinal and tumor tissue was dissected for isolation of total RNA. In each generation of *Tg(flk1:RFP)is18* fish, individuals presenting with ocular tumors were sacrificed when tumor burden reached 3 mm/25 mg of total body weight, or by 1 year of age, whichever endpoint was first reached.

### Isolation of transgenic line *Tg(flk1:RFP)is18*

The endothelial specific membrane targeted RFP reporter construct *flk1:RFP-CAAX* was assembled using standard PCR cloning methods. The zebrafish promoter for the *flk1* gene was amplified from WIK genomic DNA. The construct was cloned into the mini*pTol2* transgenesis vector [35]. Transgenics were isolated by co-injection of *in vitro* transcribed, capped, polyadenylated *Tol2* transposase mRNA [35] and the pTol2<flk1:RFP-CAAX> construct into 1 cell zebrafish WIK embryos, as described previously [36]. Three independent lines expressing RFP in the endothelial cells of the vasculature were isolated. Line *Tg(flk1:RFP)is18* contains a high copy number array Tol2<flk1:RFP-CAAX>. The *Tg(flk1:RFP)is18* line is available upon request.

### *Tol2<flk1:RFP>is18* transgene integration site cloning

A custom RNA bait library (Agilent) was used for capture of the *Tol2<flk1:RFP>* transgene concatemer and flanking genomic DNA sequences following the Agilent SureSelect Targeted Capture protocol. Briefly, a panel of overlapping biotin-labeled RNA baits (S1 Table) complementary to the *pTol2<flk1:RFP>* construct was synthesized. Genomic DNA from 5 samples was subjected to shearing, hybridization capture, library amplification, and index barcoding as outlined in the Custom SureSelect Target Enrichment protocol (Agilent). Genomic DNA was isolated from muscle tissue from one *Tol2<flk1:RFP>is18/+* heterozygous adult from the F2, F3 and F4 generation of the *is18* pedigree was isolated. Genomic DNA was also isolated from the tumor tissue from the F3 and F4 individuals. The genomic DNA was isolated with the Agilent SureSelect gDNA Extraction kit (Agilent). 3 µg of genomic DNA from each of the 5 samples was sheared to ∼250 bp (Covaris, Inc., Woburn, MA). The SureSelect Captured Libraries were 75 bp paired end multiplex sequenced in one lane on an Illumina GA II instrument at the Iowa State University DNA Facility. The number of reads per sample was: Sample 1, 1519937; Sample 2, 4790568; Sample 3, 6396525; Sample 4, 10262541; Sample 5, 23644059. Each pair of PE fastq files was aligned using GSNAP [37] to the *Danio rerio* reference genome (Zv9 64) amended with the *pTol2<flk1:RFP>* construct sequence as a separate scaffold. Reads were filtered to remove those that failed to map to both the transgene and a unique location in the zebrafish genome. The remaining reads were used to identify 1000 bp intervals in the genome that had a higher level of mapped reads than expected by random chance using a modification of a previously published bootstrap method written in the R programming language [38]. 4 locations were identified in which paired end sequences had one end mapped to the *pTol2<flk1:RFP>* construct and the other end mapped to a unique, non-repetitive sequence in the genome (S3 Table). Each site was tested for confirmation by PCR amplification of the predicted transgene-chromosome junction fragment with primers complementary to the *pTol2<flk1:RFP>* construct and the flanking genomic DNA (S4 Table). Only one of the 4 sites, chromosome 3∶24,212,813–24,212,885, was validated by amplification in all 5 samples (S1 B, C Figure). A total of 81 sequences in the 5 samples mapped to the transgene and to genomic DNA flanking either the 5′ or 3′ sides of the integration site at 24,212,944 on chromosome 3 (S1 D Figure).

### Genomic Southern blot analyses

Genomic DNA was isolated from adult zebrafish using a Qiagen midiprep kit and chemiluminescent non-radioactive Southern blot analyses performed as described previously [39]. Sequences of primers for probes for genomic Southern analyses: 450 bp probe complementary to chr 3 position 24,214,849–24,215,303; forward 5′ CTCATTCTGTCCATGTGTTCACAG 3′, reverse 5′ CTTCTTGCCTGACTTTCACAGCC 3′: 450 bp probe complementary to chr 3 positions 24,211,859–24,212,364; forward 5′ CTGACAAGCAGCTGACAGATTGG 3′, reverse 5′ GGAAGTTGCTCTCATAATTCACG 3′: 450 bp probe complementary to RFP cDNA; forward 5′ CTTCAGGGCCATGTCGCTTCTG 3′, reverse 5′ CATGGAGGGCACCGTGAACAA 3′: 477 bp probe complementary to ßlactamase cDNA in pTol2 vector backbone; forward 5′ ATCAGTGAGGCACCTATCTCAGC 3′, reverse 5′ CATAACCATGAGTGATAACACTGC 3′.

### Imaging, histopathology and immunohistochemistry

Living 3 dpf embryos were anesthetized in Tricaine, mounted in 1% low melt agarose, and imaged on a Zeiss LSM700 Confocal microscope. Whole heads dissected from adult zebrafish were fixed in 10% Formalin (Fisher) or Davidson's fixative (2∶3∶1∶3 Formalin:Ethanol:Glacial Acetic Acid:Water) for 16 hr at 4°C, decalcified in Cal-Ex (Fisher) for 2 days at 4°C, then processed and embedded in paraffin at the Iowa State University Clinical Histopathology Laboratory. Paraffin blocks of head tissue were serial sectioned at 6um on a Shandon Finesse 325 microtome, stained with Hematoxylin 7211 Richard-Allan Scientific (Fisher) and 3% Eosin Y (Argos Organics), and mounted with Permount (Fisher). Sections were imaged on a Zeiss Axioskop II using Nikon Coolpix and Nikon Rebel cameras.

For immunohistochemistry heads were removed from anesthetized adults and fixed in 4% paraformaldehyde overnight at 4°C, decalcified in Cal-Ex for 2 days at 4°C, then processed for embedding in optimal cutting temperature (OCT) medium (Fisher). Heads were serial sectioned at 12 µm on a Microm HM 550 cryotstat at 25°C. For BrdU labeling experiments, adults were incubated in 5 µM BrdU (Sigma) in fish water (60.5 mg ocean salts/l) for 2 hours, placed in fresh fish water for 4 hours, then sacrificed and processed as above for immunohistochemical labeling experiments. Antibody labeling of cryosectioned tissue was as described previously [9], [40]. Dilutions and primary antibodies used for labeling sectioned tissue were as follows: rabbit polyclonal anti-recoverin 1∶1000 (Millipore); mouse monoclonal anti-glial fibrillary acid protein GFAP 1∶1000 obtained from the Zebrafish International Research Center (ZIRC); rabbit polyclonal anti-Brain Lipid Binding Protein BLBP 1∶200 (Abcam); mouse monoclonal anti-SV2 1∶100 (Developmental Studies Hybridoma Bank); mouse monoclonal anti-RT97 1∶250 (Developmental Studies Hybridoma Bank); anti-BrdU 1∶500 (Bio-Rad). Alexa-594 and Alexa-697 conjugated secondary antibodies (Invitrogen) and Cy3 and Cy5 conjugated secondary antibodies (Jackson Immunoresearch Labs) were used at a dilution of 1∶500. Tissues were counterstained with 5 µg/ml DAPI (Sigma) and mounted in Fluorogel (EMS). To aid antigen retrieval tissues were pretreated with 2 M HCl (for anti-BrdU labeling). Immunofluorescent labeling of cryosections was imaged on a Nikon Microphot-FXA microscope and captured using a QImaging Retiga 2000R Fast 1394 camera and QCapture software. All images were edited and assembled in Adobe Photoshop CS2.

### RT-PCR and in situ hybridization

Total RNA for staged developmental series, tissue panel, and analysis of homozygous embryos, was isolated with a Qiagen RNeasy Isolation Kit. RT-PCR was carried out using a One-Step RT-PCR Kit (Invitrogen). cDNA for *lincRNAis18* was amplified by RT-PCR out of total RNA isolated from wild type 48 hpf embryos or adult retina. cDNAs were cloned into pBluescript or the pCR4-TOPO vector (Invitrogen). Primers for amplification were as follows: *lincRNAis18* forward1 5′ TCACTGTCTGCTGCTGACGATC 3′, *lincRNAis18* nested forward 5′ GACAGACTCTGGCACAATCTCTG 3′, *lincRNAis18* forward2 5′ CAACAGTTTCCTGAACACGC 3′, *lincRNAis18* reverse 5′ CAACGCTTTAACAGAACAGACTTC 3′, *lincRNAis18* nested reverse 5′ TGACATACTCACATAAACTCCACGC 3′; *cbx1a* forward 5′ TCGATGAGCATGAGCCAACC 3′, *cbx1a* reverse 5′ CCAAGCCTAGTTCTTGTCATCTTTC 3′.

Primers complementary to the *ribosomal protein S6 kinase b*, *rps6kb1* gene were used as control for RT-PCR reactions. forward *rps6kb1* forward 5′ CATGGCGACGGTGCGTTCAT 3′; *rps6kb1* reverse 5′ AGCTTGCCGCCCGTCTGAAA 3′. Digoxygenin-labeled antisense and sense probes for in situ hybridization were synthesized using the T3 Dig labeled RNA synthesis kit (Roche). In situ hybridization on 12–16 µm cryosections of head and eye tissue was performed as described [41]. Probes were purified over Biorad Biospin or Qiagen RNeasy MinElute Cleanup Kit columns following the manufacturers instructions and stored in 50% formamide at −20°C.

### TALEN directed isolation of *lincRNAis18* deletion allele

TALEN pairs targeting sequences in exon 2 and exon 5 of lincRNAis18 were designed using TAL Effector-Nucleotide Targeter 2.0 [42] and synthesized using the modified GoldyTALEN scaffold [43]. 1-cell stage WIK embryos were co-injected with 30 or 60 pg *in vitro* transcribed TALEN mRNA targeting sequences in exons two and exons five of *lincRNAis18*. Individual injected embryos were assayed for mutation efficiency by disruption of restriction enzyme sites in an amplicon spanning the targeted region. Genomic DNA was extracted from embryos and adult fin clips by placing tissue in 50 µl 50 mM NaOH and heating at 95°C for 10 minutes. Primers to amplify *lincRNAis18* exon two: exon2F 5′ GGTCATGTCCTTGTGTTTTG 3′; exon2R′ CTCCAGCTCCTGTGTATTCATTG 3′. Primers to amplify *lincRNAis18* exon five: exon5F 5′ CCACAAGTTTCATGTGGCTCT 3′; exon5R 5′ TGGATTACTCGTAACTGAGGAAAAAC 3′. Founders were raised to adulthood and screened for germline transmission of the deletion allele. 20 individual embryos from 27 F0 adults were tested by PCR amplification across the exon two – exon five junction using primers exon2F 5′ GGTCATGTCCTTGTGTTTTG 3′ and exon5R 5′ TGGATTACTCGTAACTGAGGAAAAAC 3′.

### RNA-Seq, real time PCR and differential gene expression analyses

RNA-Seq data are available in the ArrayExpress database (www.ebi.ac.uk/arrayexpress) under accession number E-MTAB-2886. Tissues for isolation of total RNA for RNA-Seq libraries were dissected from three age-matched genotypes. Total RNA was isolated using Qiagen RNeasy RNA Isolation Kit (Qiagen). The first sample, “Wild Type”, contained three pooled retinas from 6-month-old wild type adults. The second sample, “Pretumor”, consisted of three pooled retinas from age-matched heterozygous *Tg(flk1:RFP)is18*/+ adults that did not show obvious gross ocular tumors. The third sample, “Tumor”, consisted of tumor tissue dissected from the eyes of two age-matched heterozygous *Tg(flk1:RFP)is18*/+ adults that had advanced, large tumors that filled the vitreous and distorted the ocular cavity. RNA-Seq libraries from each sample were 100 bp paired-end sequenced in individual lanes on an Illumina HiSeq 2000 instrument at the Genome Sequencing and Analysis Core Resource, Duke Institute for Genome Sciences and Policy, Duke University. Mapping of the RNA-Seq data was performed at the Genome Informatics Facility, Iowa State University. To map the RNA-Seq data the *Danio rerio* reference genome v9 and gene models (release 71) were downloaded from ensemble. Sequences were mapped using GSNAP version 2012-07-20 [37]. Raw read counts per gene were determined using the program HTSeq-count (http://www-huber.embl.de/users/anders/HTSeq/) [44] to identify unique reads that mapped within a gene model. Upper quartile normalization was applied to the raw reads across the three samples. The Fisher's exact test was performed to determine differential expression of a gene between samples. Q-value estimation for false discovery rate was performed in R using open source software qvalue at bioconductor (http://bioconductor.org/biocLite.R; Alan Dabney, John D. Storey and with assistance from Gregory R. Warnes. qvalue: Q-value estimation for false discovery rate control. R package version 1.32.0). Heat maps representing log_2_(fold change) in gene expression were created in Excel. GO Term analysis was done at the Gene Ontology (GO) Tools website http://go.princeton.edu.

Two-Step real time PCR was performed on a Roche LightCycler 480 instrument using SYBR green reaction mix (Fisher). RNA isolation from Wild Type, Pre-tumor, and Tumor tissues was as described above. cDNA template was synthesized with SuperScript II (Invitrogen). qRT-PCR reactions were run in triplicate for each tissue template and primer pair. Primers for qRT-PCR are listed in S4 Table.

## Supporting Information

S1 Figure **Molecular mapping of the** ***Tol2<flk1:RFP>*** **concatemer transgene in zebrafish line** ***Tg(flk1:RFP)is18*** **to chromosome 3.** (A) Schematic of Agilent Sure Select Target Enrichment mapping technique. *Tg(flk1:RFP)is18* and flanking genomic sequences were captured with complementary biotin-RNA probes followed by Illumina GAIIx sequencing of barcoded libraries and mapping to the zebrafish genome (B) Snapshot of alignment to chromosome 3 in the zebrafish genome of genomic DNA-transgene junction fragments captured with a custom SureSelect Target Enrichment kit. (C) Diagram illustrating integration site of the *Tol2<flk1:RFP>* concatemer at position 24, 212, 944 on chromosome 3. The sequence flanking the transgene, containing an 8 bp duplication at the integration site, is shown below. Primers used for PCR amplification of the junction fragments at the integration site are shown. Chr3F and chr3R, position of primers on chromosome 3. Tol2-5′R and Tol2-3′F sit within the left and right inverted repeats of the *Tol2<flk1:RFP>* transposon. (D) PCR products verify the location of the transgene integration in the 5 genomic samples used for SureSelect Target Enrichment. *, amplification of the 350 bp 5′ genomic-transgene junction fragment. **, amplification of the 200 bp 3′ genomic-transgene junction fragment.
(TIF)

S2 Figure **RFLP caused by integration of** ***Tol2<flk1:RFP>*** **concatemer at position 24, 212, 944 on chromosome 3.** (A) Diagram of *Tol2<flk1:RFP>* transposon construct with position of probe complementary to RFP cDNA (red box). (B) *BamH*I/*Bgl*II restriction map of region surrounding *Tol2<flk1:RFP>* concatemer integration on chromosome 3. Blue box shows position of probe complementary to region on chromosome 3 just 5′ to integration site. (C) Genomic Southern blots of BamH1/BglII double digested genomic DNA from wild type WIK, 6^th^ generation *Tg(flk1:RFP)is18*, and 6^th^ generation non-transgenic +/+ siblings. BamHI cuts once within the *Tol2<flk1:RFP>* transposon, releasing each copy from the concatemer. Left panel shows chromosome 3 RFLP due to transgene integration (blue asterisk) present only in *Tg(flk1:RFP)is18* transgenic fish. Right panel shows an intense band at the expected size for the *Tol2<flk1:RFP>* transposon construct and many other bands of varying sizes. This confirms the identity of the *Tg(flk1:RFP)is18* transgenic fish and reveals the complex nature and disorganization of the transgenes in the high copy number array.
(TIF)

S3 Figure **RFLP caused by integration of** ***Tol2<flk1:RFP>*** **concatemer at position 24, 212, 944 on chromosome 3.** (A) Diagram of *Tol2<flk1:RFP>* transposon construct with position of probe complementary to ßlactamase cDNA (orange box) in the vector backbone. (B) *BstE*II restriction map of region surrounding *Tol2<flk1:RFP>* concatemer integration on chromosome 3. *BstE*III does not cut in the *Tol2<flk1:RFP>* concatemer. Blue box shows position of probe complementary to region on chromosome 3 just 5′ to integration site. (C) Genomic Southern blots of *BstE*III digested genomic DNA from wild type WIK, 5^th^ generation *Tg(flk1:RFP)is18*, 6^th^ generation *Tg(flk1:RFP)is18*, and 6^th^ generation non-transgenic +/+ siblings. Left panel shows blot of linear digested plasmids of known size for comparison. Panel second from left shows high molecular weight band (blue asterisk) corresponding to chromosome 3 RFLP caused by concatemer integration. Right panels show blots hybridized with a probe specific to the transgene construct in the concatemer. The intense band (orange asterisk) corresponds to the concatemer integrated in chromosome 3. The band runs at the same position as the band recognized by the chromosome 3 probe. Far right panel represents longer exposure of blot shown in second panel from right.
(TIF)

S4 Figure **STR marker linkage analysis of the** ***Tol2<flk1:RFP>*** **concatemer in line** ***Tg(flk1:RFP)is18*** **.** Upper panel. Genomic position and name of Short Tandem Repeat markers in the region of the transgene integration site on chromosome 3. Representative images of marker PCR products show genotype of an F5 generation *Tg(flk1:RFP)is18* and a wild type WIK fish used for linkage analysis. Lower panel. Analysis in 20 offspring from a cross between the genotyped *Tg(flk1:RFP)is18* and wild type WIK adults shows linkage of the chromosome to the long allele of Z7419, the short allele of G39247, the long allele of Z5197, and the long allele of cbx1aSTS. Further analyses of 200 progeny (Table 1) confirmed tight linkage of the concatemer integration site to the Z7419, G3927, Z5197 and cbx1aSTS markers.
(TIF)

S5 Figure **Examination of zebrafish** ***lincRNAis18*** **expression in early development and adult tissues.** (A) Nested RT-PCR showing expression of *lincRNAis18* within the adult zebrafish retina. (B) In situ hybridization using non-radioactive DIG-labeled *lincRNAis18* probes on adult zebrafish retina cryosections. *lincRNAis18* expression is detected in the ganglion cell layer (GCL) and a subset of cells at the vitreal side of the inner nuclear layer (INL) (left, middle). Negative control, *lincRNAis18* sense DIG-labeled probe (right). (C, D) RT-PCR showing relative levels of *lincRNAis18* expression throughout development and in adult tissues. Control, expression of ribosomal protein S6 kinase b, polypeptide 1, *rps6kb1*. Blue bracket and asterisks indicate *lincRNAis18* cloned and sequence confirmed products. Red bracket and asterisks indicate nonspecific products cloned and confirmed by sequencing. GCL, ganglion cell layer; INL, inner nuclear layer; ONL, outer nuclear layer; RPE, retinal pigmented epithelium. Scale bars 100 µm.
(TIF)

S6 Figure **Genotyping of progeny from** ***Tg(flk1:RFP)is18*** **incross.** (A–C) Five individual larvae from each progeny class were genotype confirmed by PCR. Primer pair 1, chr3F and chr3R, amplify a fragment of the wild type chromosome 3 spanning the concatemer integration site. Primer pair 2, chr3F and Tol2R, amplify a genomic DNA-transgene junction fragment. Primer pair 3, control primers for amplification of a fragment of the *flh* gene. (A) Wild type +/+ sibling larvae. As expected primer pair 1 amplifies the wild type fragment of chromosome 3, while the concatemer genomic junction fragment that would be amplified by primer pair 2 is absent. (B) Homozygous mutant *Tg(flk1:RFP)is18*/*Tg(flk1:RFP)is18* larvae. As expected, the wild type fragment of chromosome 3 is absent, while the concatemer genomic DNA junction fragment is present. (C) Heterozygous *Tg(flk1:RFP)is18*/+ genotyped larvae. Both the wild type chromosome 3 fragment and the concatemer genomic DNA junction fragment amplify.
(TIF)

S7 Figure **TALEN directed mutagenesis of** ***lincRNAis18*** **exons two and five.** (A) TALEN pairs targeting exon 2 and exon 5 of *lincRNAis18*. TALEN spacers are shown in red./marks location of FOK1 endonuclease cut site. *Hinc*II restriction enzyme site (exon 2) and *Mse*I restriction enzyme site (exon 5) are underlined. (B) Individual embryos injected with TALENs targeting *lincRNAis18* exon 2 (left panel) or exon 5 (right panel). The presence of *Hinc*II and *Mse*I digestion resistant amplicons demonstrates mutation of the targeted site.
(TIF)

S8 Figure **The** ***lincRNAis18^e2e5del^*** **deletion allele is homozygous viable.** (A) Diagram of *lincRNAis18* gene structure and exon 2- exon 5 deletion allele. Primers e2F and e2R flank exon 2; primers e5F and e5R amplify exon 5. The genetic marker G38247 is located between exons 2 and 3. The genetic marker Z7419 is located 1 Mb downstream of the 3′ end of *lincRNAis18*. (B) Genotyping of fin clips from 10 adult progeny of a *lincRNAis18^e2e5del^/+* incross. Genomic DNA was amplified with primer pairs listed. The exon 2- exon 5 fusion amplicon was detected in 4/10 adults, indicating they were homozygous for the *lincRNAis18^e2e5del^* chromosome (top panel). As expected these 4 adults lacked the G39247 marker (4^th^ panel).
(TIF)

S9 Figure **Loss of** ***lincRNAis18*** **mRNA at polyA selection step during RNA-Seq library preparation.** RT-PCR shows presence of *cbx1a* and *lincRNAis18* in the total RNA from wild type retina sample used for RNA-Seq cDNA library preparation. The *cbx1a* transcript was detected following a single round of RT-PCR amplification in the total RNA sample (T) and in the Illumina RNA-Seq cDNA library sample (L). The *lincRNAis18.v1* and.v2 transcripts were present in the total RNA sample (blue asterisks). However, only nonspecific products amplified from the cDNA library sample after one round of RT-PCR (red asterisks). Nested PCR resulted in amplification of multiple alternatively spliced transcripts from both samples (Right panel, blue asterisks). Bands marked with asterisks were cloned and sequence verified.
(TIF)

S1 Table **SureSelect Target Enrichment Antisense 120nt Bait Library tiled across pTol2<flk1:RFP> plasmid vector.**
(XLS)

S2 Table **Ilumina Paired End Reads from Custom SureSelect Target Enrichment of** ***Tol2<flk1:RFP>is18*** **transgene integration site.**
(XLSX)

S3 Table **Candidate** ***Tol2<flk1:RFP>is18*** **transgene integration locations.**
(XLSX)

S4 Table **Primers for genotyping and qRT-PCR.**
(XLSX)

S5 Table **Alternatively spliced isoforms of** ***lincRNAis18*** **RT-PCR products.**
(XLSX)

S6 Table **Illumina Hi-Seq RNA-Seq data from zebrafish wild type retina,** ***Tg(flk1:RFP)is18/+*** **pretumor retina and** ***Tg(flk1:RFP)is18/+*** **tumor tissue.**
(XLSX)

S7 Table **All mapped reads from RNA-Seq of wild type,** ***Tg(flk1:RFP)is18/+*** **pretumor and** ***Tg(flk1:RFP)is18/+*** **tumor retinal tissue.**
(XLSX)

S8 Table **Differential expression of genes required for photoreception and neurotransmission in** ***Tg(flk1:RFP)is18/+*** **pre-tumor retina and tumor tissue.**
(XLSX)

S9 Table **Differential Expression of lncRNAs in** ***Tg(flk1:RFP)is18/+*** **pre-tumor retina and tumor tissues.**
(XLSX)

S1 Checklist (PDF)
