# Supplementary figures and images for: Molecular and Cellular Characterization of a Zebrafish Optic Pathway Tumor Line Implicates Glia-Derived Progenitors in Tumorigenesis
Source: PLoS One. 2014 Dec 8;9(12):e114888. doi: 10.1371/journal.pone.0114888 (PMC4259487; doi:10.1371/journal.pone.0114888)

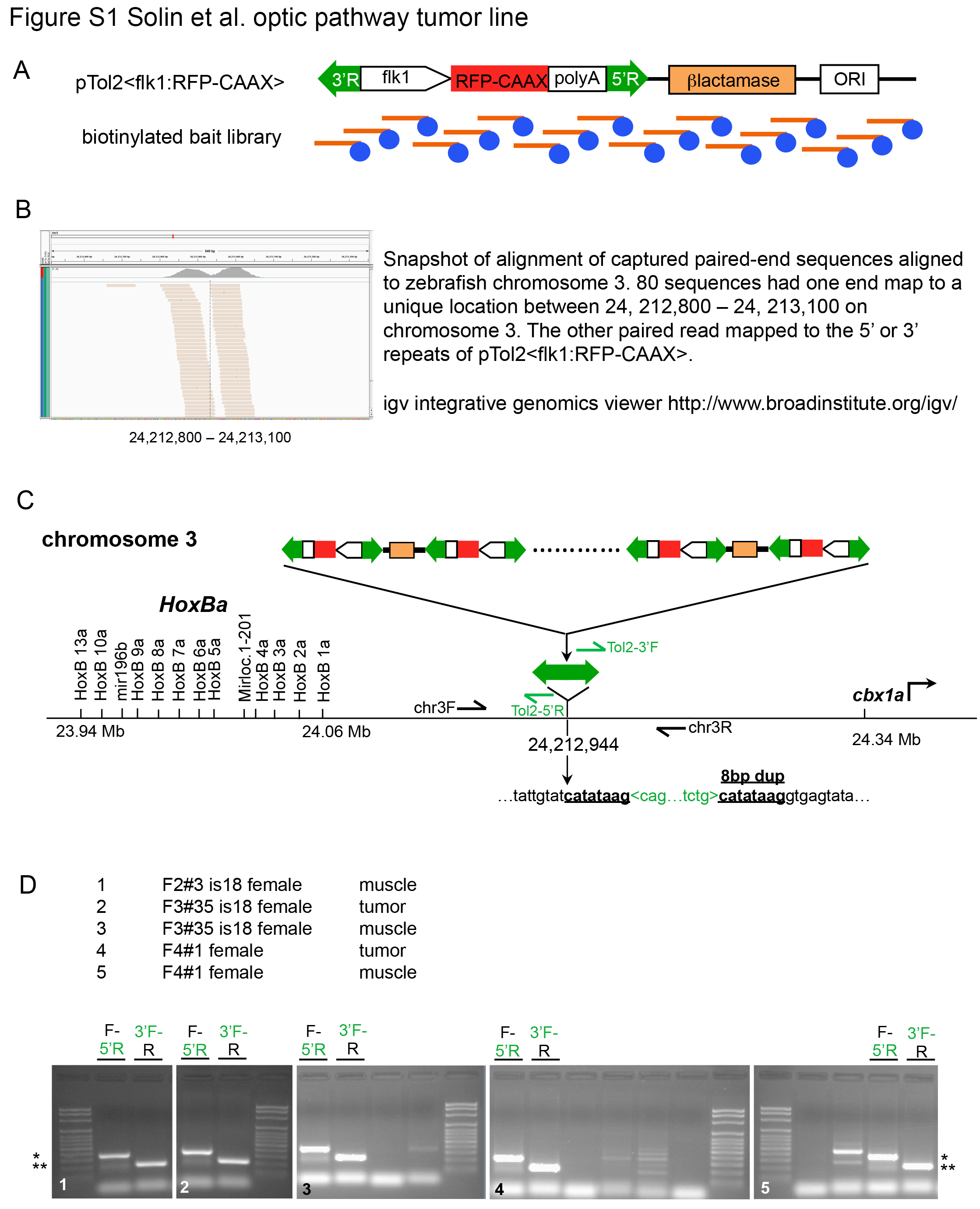

Supplement: S1 Figure — Molecular mapping of the Tol2 concatemer transgene in zebrafish line Tg(flk1:RFP)is18 to chromosome 3. (A) Schematic of Agilent Sure Select Target Enrichment mapping technique. Tg(flk1:RFP)is18 and flanking genomic sequences were captured with complementary biotin-RNA probes followed by Illumina GAIIx sequencing of barcoded libraries and mapping to the zebrafish genome (B) Snapshot of alignment to chromosome 3 in the zebrafish genome of genomic DNA-transgene junction fragments captured with a custom SureSelect Target Enrichment kit. (C) Diagram illustrating integration site of the Tol2 concatemer at position 24, 212, 944 on chromosome 3. The sequence flanking the transgene, containing an 8 bp duplication at the integration site, is shown below. Primers used for PCR amplification of the junction fragments at the integration site are shown. Chr3F and chr3R, position of primers on chromosome 3. Tol2-5′R and Tol2-3′F sit within the left and right inverted repeats of the Tol2 transposon. (D) PCR products verify the location of the transgene integration in the 5 genomic samples used for SureSelect Target Enrichment. *, amplification of the 350 bp 5′ genomic-transgene junction fragment. **, amplification of the 200 bp 3′ genomic-transgene junction fragment. (TIF) [file pone.0114888.s001.tif]

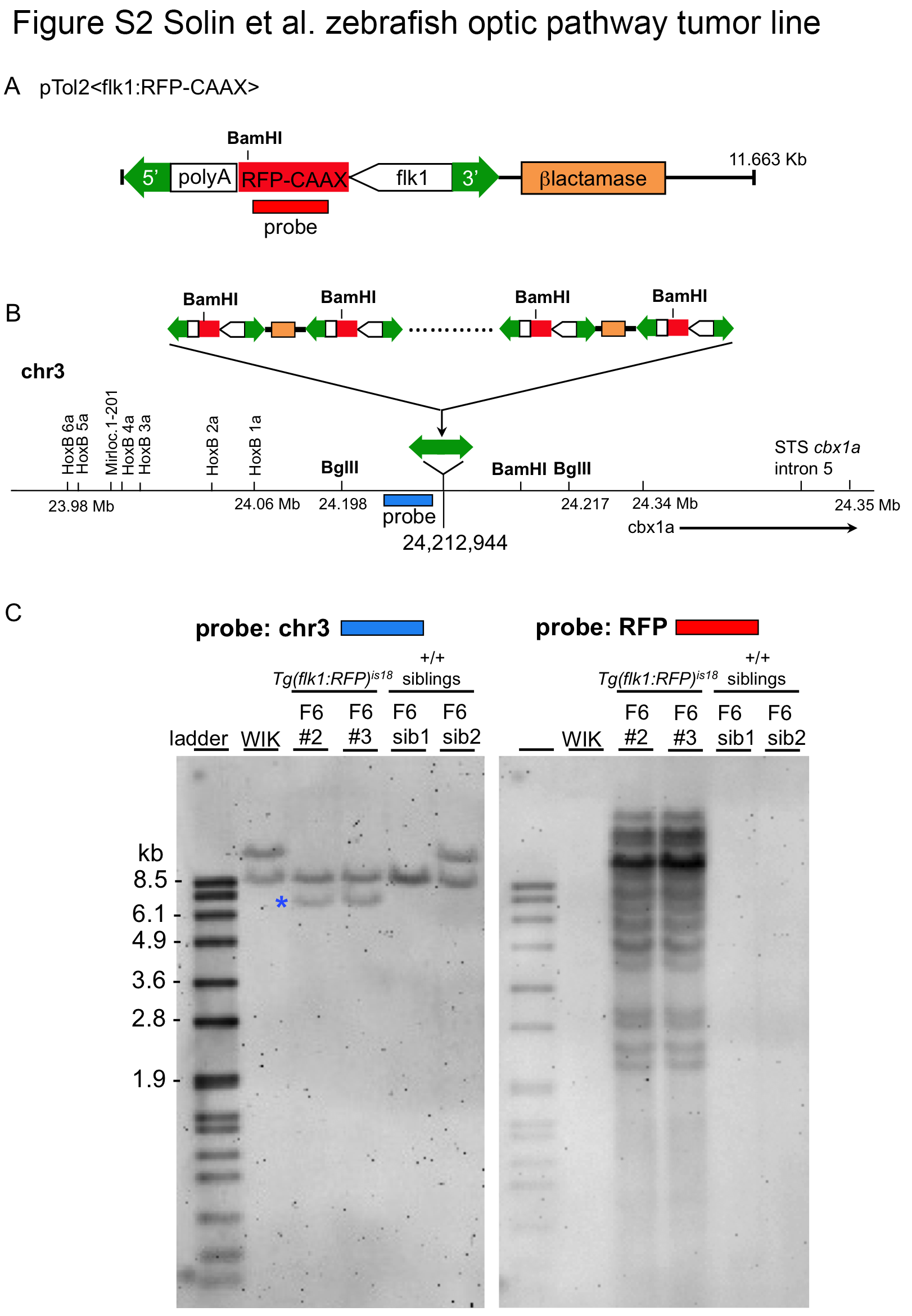

Supplement: S2 Figure — RFLP caused by integration of Tol2 concatemer at position 24, 212, 944 on chromosome 3. (A) Diagram of Tol2 transposon construct with position of probe complementary to RFP cDNA (red box). (B) BamHI/BglII restriction map of region surrounding Tol2 concatemer integration on chromosome 3. Blue box shows position of probe complementary to region on chromosome 3 just 5′ to integration site. (C) Genomic Southern blots of BamH1/BglII double digested genomic DNA from wild type WIK, 6th generation Tg(flk1:RFP)is18, and 6th generation non-transgenic +/+ siblings. BamHI cuts once within the Tol2 transposon, releasing each copy from the concatemer. Left panel shows chromosome 3 RFLP due to transgene integration (blue asterisk) present only in Tg(flk1:RFP)is18 transgenic fish. Right panel shows an intense band at the expected size for the Tol2 transposon construct and many other bands of varying sizes. This confirms the identity of the Tg(flk1:RFP)is18 transgenic fish and reveals the complex nature and disorganization of the transgenes in the high copy number array. (TIF) [file pone.0114888.s002.tif]

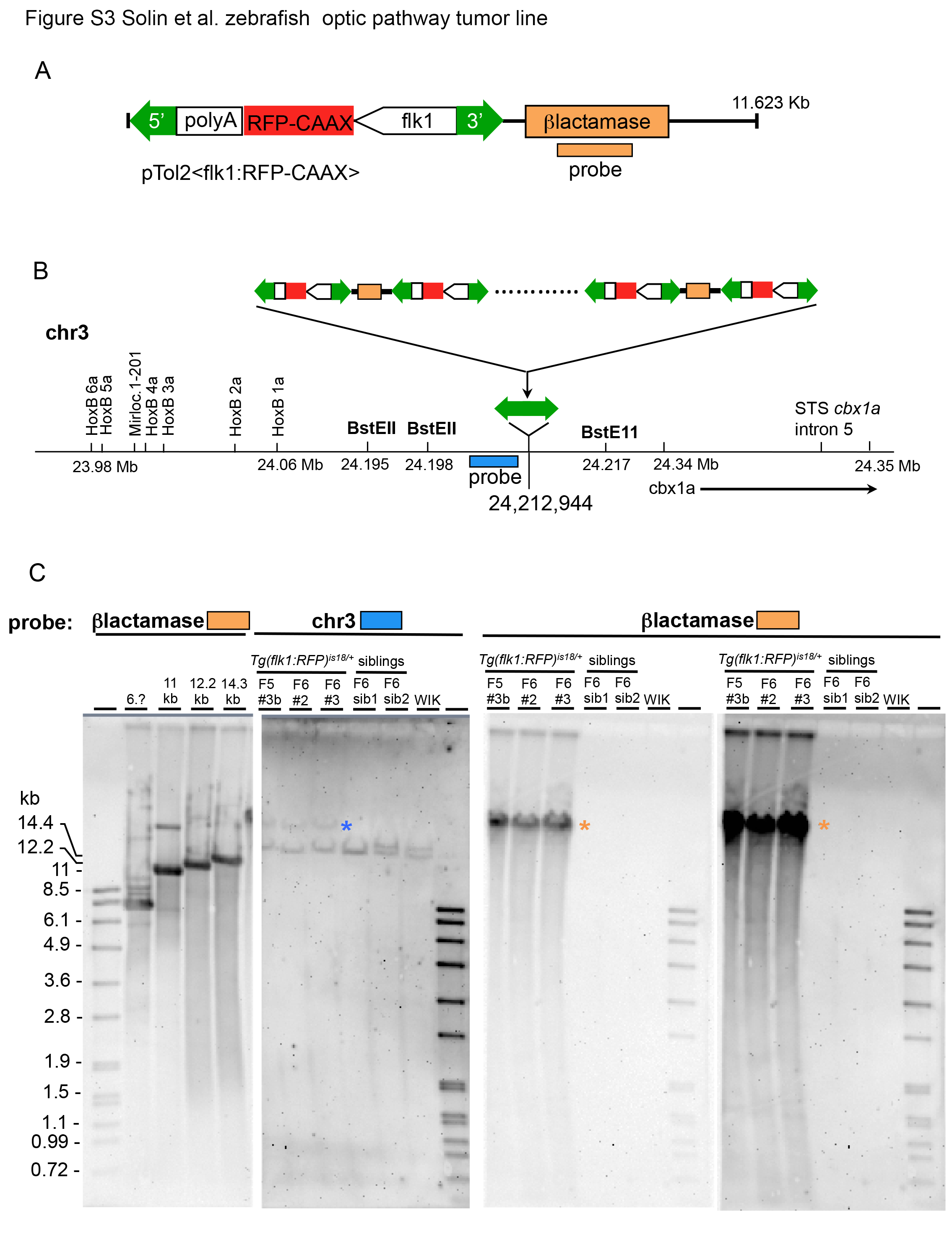

Supplement: S3 Figure — RFLP caused by integration of Tol2 concatemer at position 24, 212, 944 on chromosome 3. (A) Diagram of Tol2 transposon construct with position of probe complementary to ßlactamase cDNA (orange box) in the vector backbone. (B) BstEII restriction map of region surrounding Tol2 concatemer integration on chromosome 3. BstEIII does not cut in the Tol2 concatemer. Blue box shows position of probe complementary to region on chromosome 3 just 5′ to integration site. (C) Genomic Southern blots of BstEIII digested genomic DNA from wild type WIK, 5th generation Tg(flk1:RFP)is18, 6th generation Tg(flk1:RFP)is18, and 6th generation non-transgenic +/+ siblings. Left panel shows blot of linear digested plasmids of known size for comparison. Panel second from left shows high molecular weight band (blue asterisk) corresponding to chromosome 3 RFLP caused by concatemer integration. Right panels show blots hybridized with a probe specific to the transgene construct in the concatemer. The intense band (orange asterisk) corresponds to the concatemer integrated in chromosome 3. The band runs at the same position as the band recognized by the chromosome 3 probe. Far right panel represents longer exposure of blot shown in second panel from right. (TIF) [file pone.0114888.s003.tif]

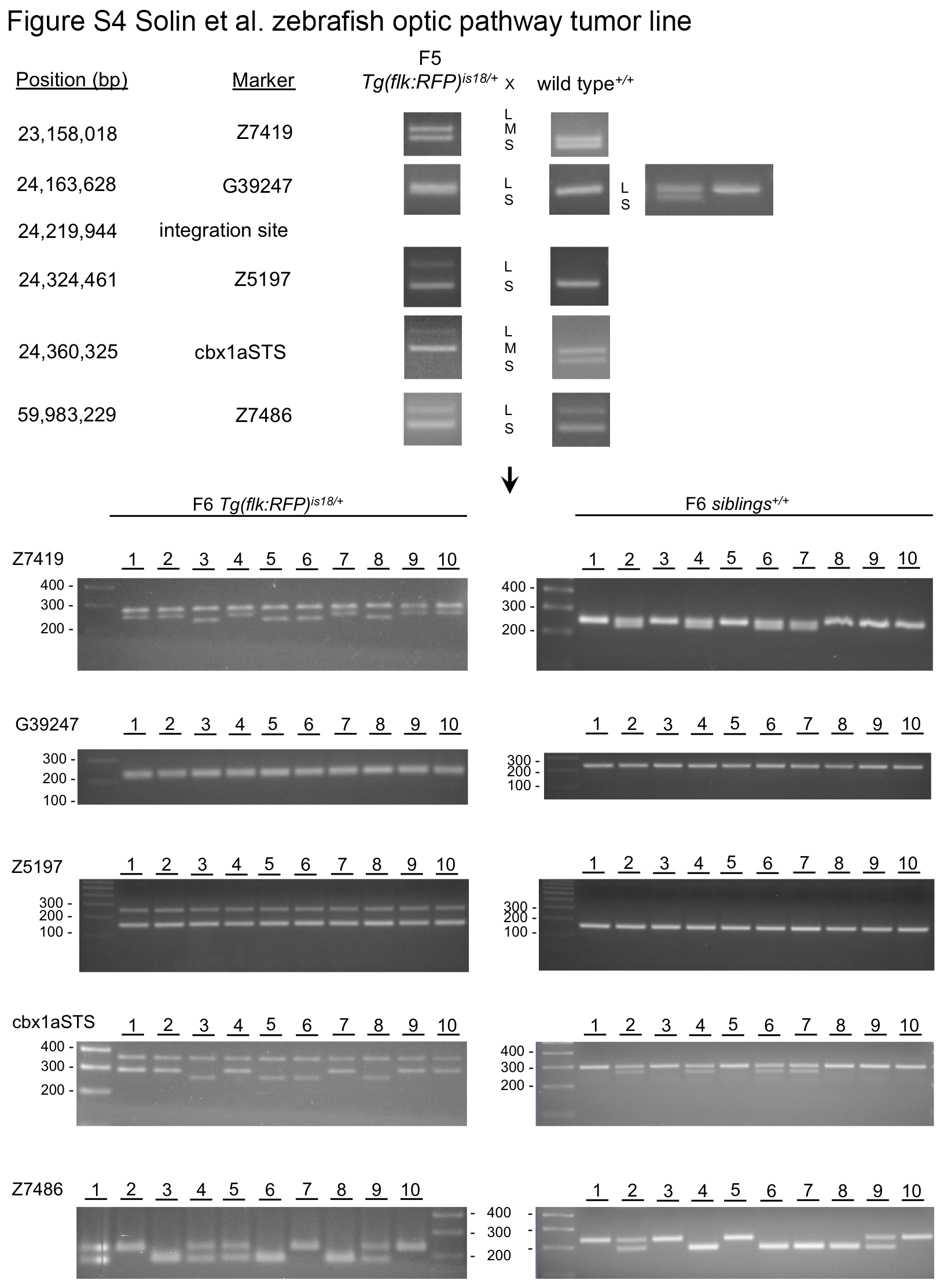

Supplement: S4 Figure — STR marker linkage analysis of the Tol2 concatemer in line Tg(flk1:RFP)is18 . Upper panel. Genomic position and name of Short Tandem Repeat markers in the region of the transgene integration site on chromosome 3. Representative images of marker PCR products show genotype of an F5 generation Tg(flk1:RFP)is18 and a wild type WIK fish used for linkage analysis. Lower panel. Analysis in 20 offspring from a cross between the genotyped Tg(flk1:RFP)is18 and wild type WIK adults shows linkage of the chromosome to the long allele of Z7419, the short allele of G39247, the long allele of Z5197, and the long allele of cbx1aSTS. Further analyses of 200 progeny (Table 1) confirmed tight linkage of the concatemer integration site to the Z7419, G3927, Z5197 and cbx1aSTS markers. (TIF) [file pone.0114888.s004.tif]

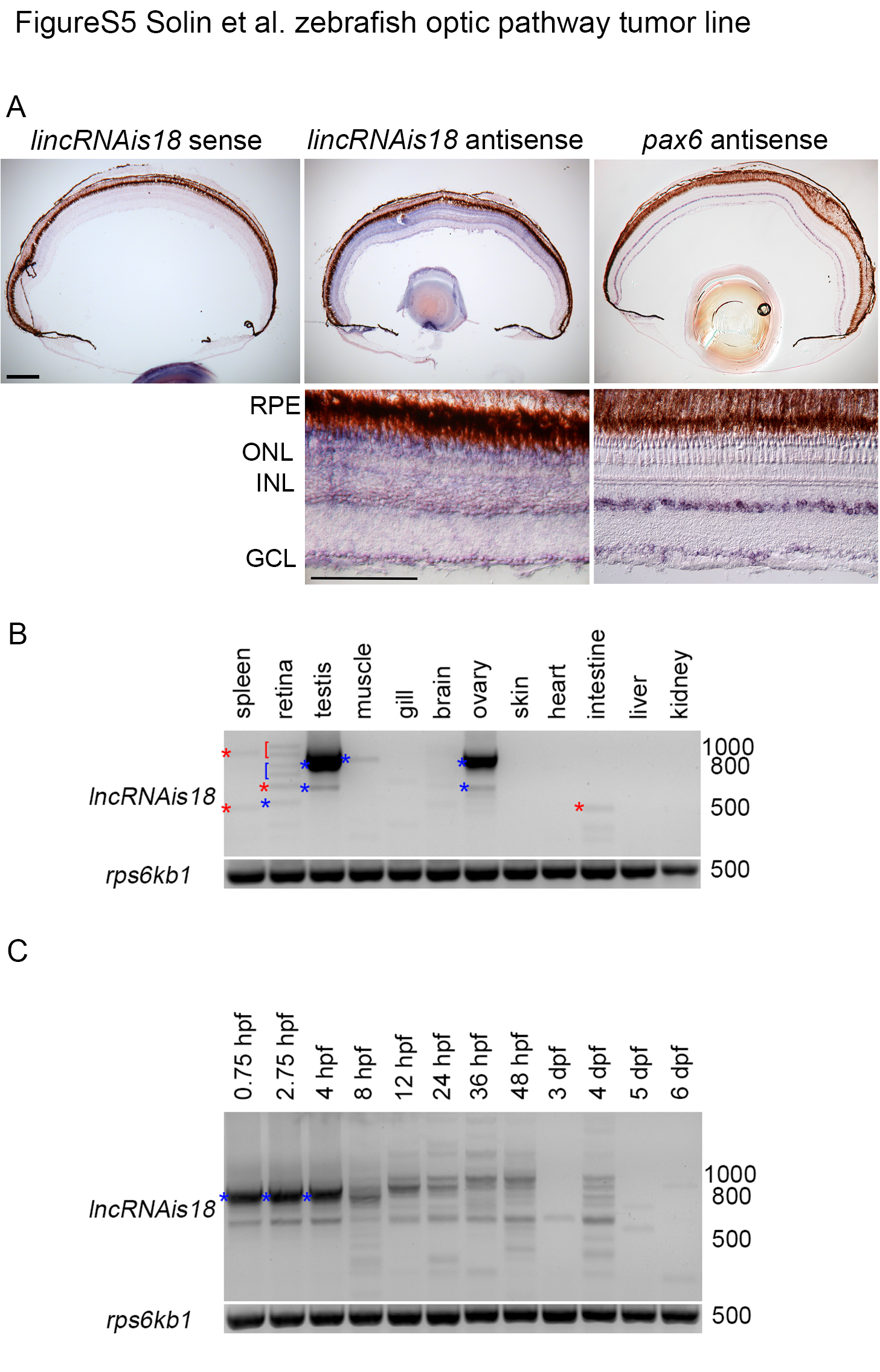

Supplement: S5 Figure — Examination of zebrafish lincRNAis18 expression in early development and adult tissues. (A) Nested RT-PCR showing expression of lincRNAis18 within the adult zebrafish retina. (B) In situ hybridization using non-radioactive DIG-labeled lincRNAis18 probes on adult zebrafish retina cryosections. lincRNAis18 expression is detected in the ganglion cell layer (GCL) and a subset of cells at the vitreal side of the inner nuclear layer (INL) (left, middle). Negative control, lincRNAis18 sense DIG-labeled probe (right). (C, D) RT-PCR showing relative levels of lincRNAis18 expression throughout development and in adult tissues. Control, expression of ribosomal protein S6 kinase b, polypeptide 1, rps6kb1. Blue bracket and asterisks indicate lincRNAis18 cloned and sequence confirmed products. Red bracket and asterisks indicate nonspecific products cloned and confirmed by sequencing. GCL, ganglion cell layer; INL, inner nuclear layer; ONL, outer nuclear layer; RPE, retinal pigmented epithelium. Scale bars 100 µm. (TIF) [file pone.0114888.s005.tif]

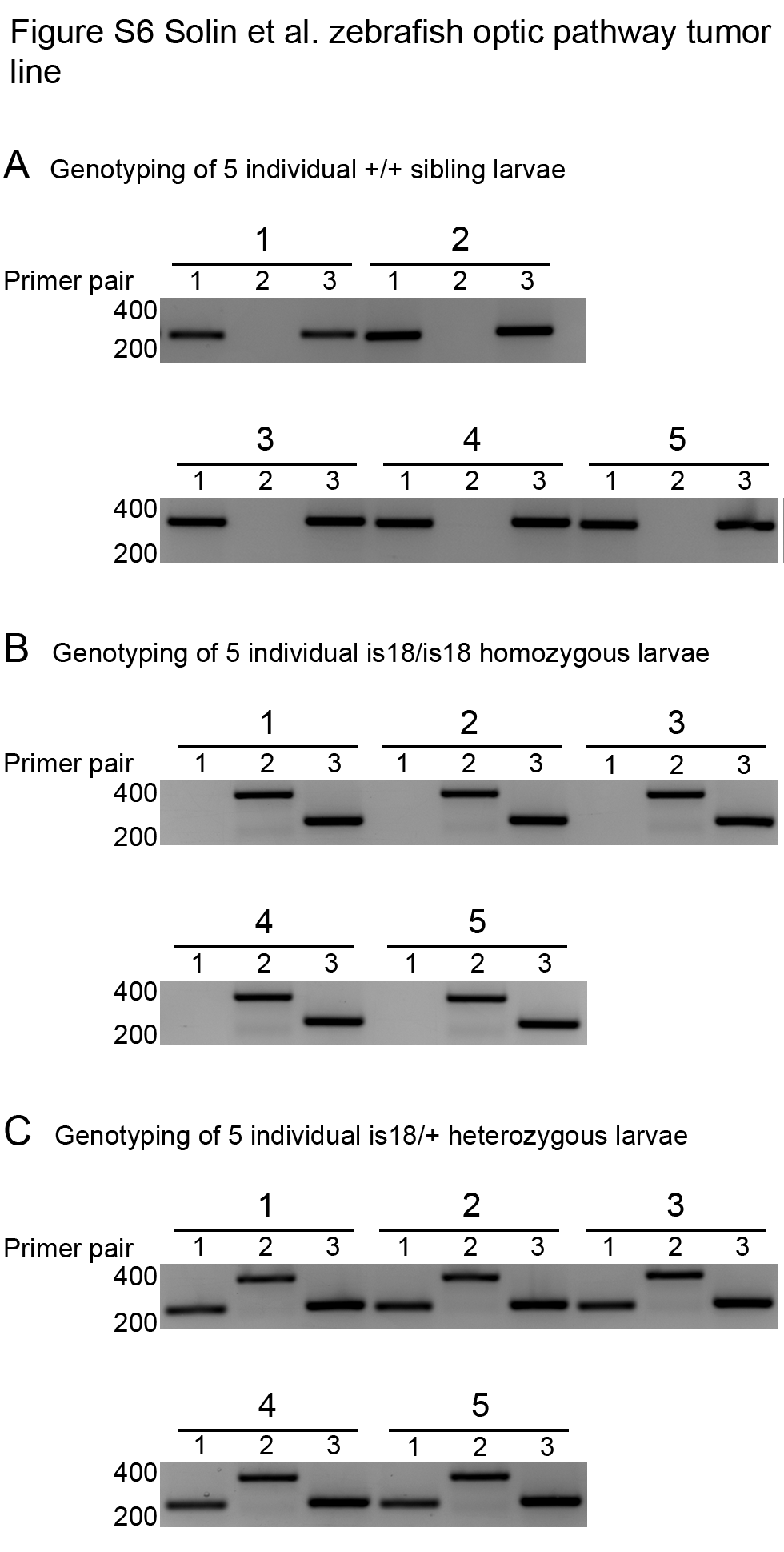

Supplement: S6 Figure — Genotyping of progeny from Tg(flk1:RFP)is18 incross. (A–C) Five individual larvae from each progeny class were genotype confirmed by PCR. Primer pair 1, chr3F and chr3R, amplify a fragment of the wild type chromosome 3 spanning the concatemer integration site. Primer pair 2, chr3F and Tol2R, amplify a genomic DNA-transgene junction fragment. Primer pair 3, control primers for amplification of a fragment of the flh gene. (A) Wild type +/+ sibling larvae. As expected primer pair 1 amplifies the wild type fragment of chromosome 3, while the concatemer genomic junction fragment that would be amplified by primer pair 2 is absent. (B) Homozygous mutant Tg(flk1:RFP)is18/Tg(flk1:RFP)is18 larvae. As expected, the wild type fragment of chromosome 3 is absent, while the concatemer genomic DNA junction fragment is present. (C) Heterozygous Tg(flk1:RFP)is18/+ genotyped larvae. Both the wild type chromosome 3 fragment and the concatemer genomic DNA junction fragment amplify. (TIF) [file pone.0114888.s006.tif]

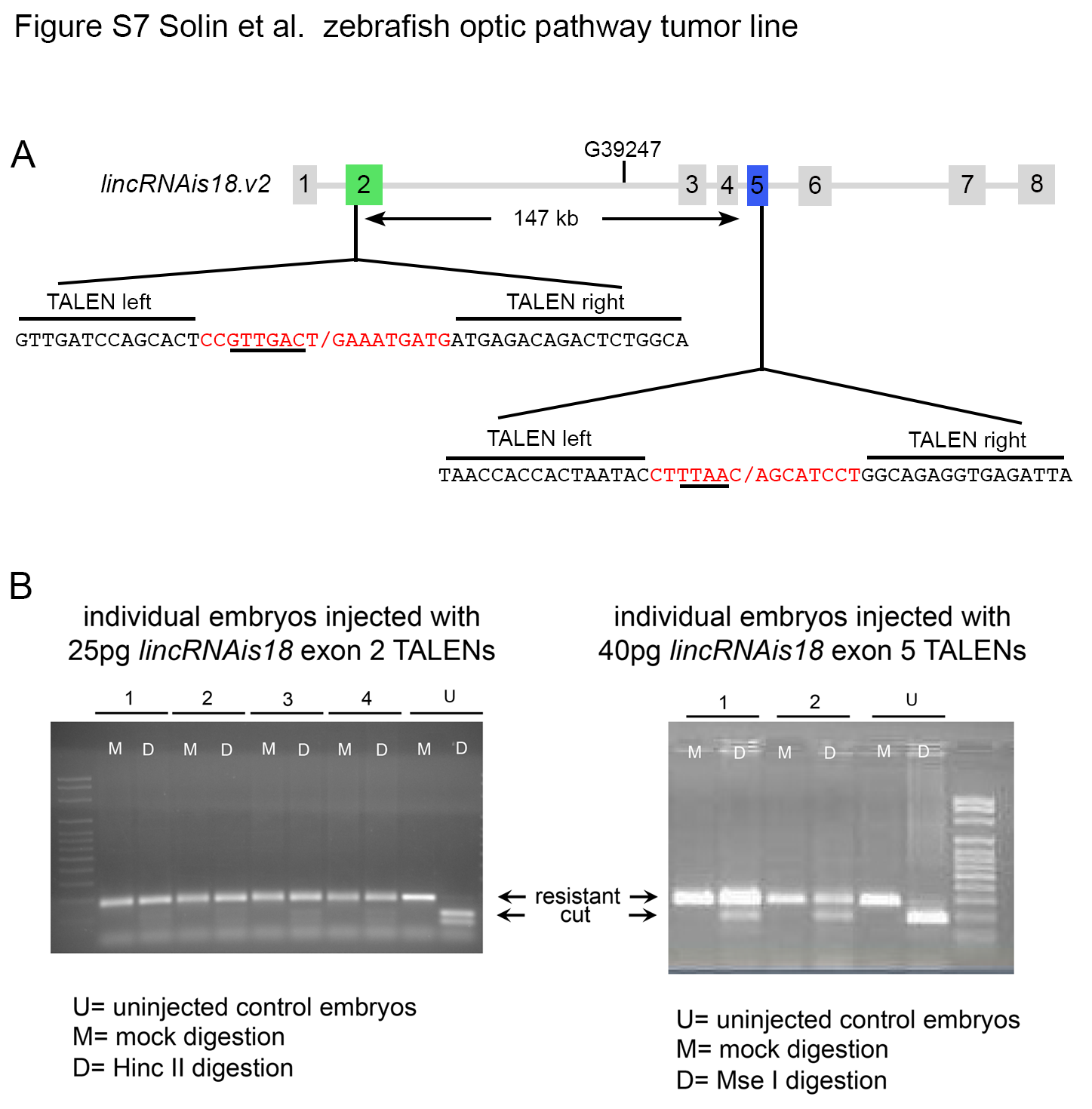

Supplement: S7 Figure — TALEN directed mutagenesis of lincRNAis18 exons two and five. (A) TALEN pairs targeting exon 2 and exon 5 of lincRNAis18. TALEN spacers are shown in red./marks location of FOK1 endonuclease cut site. HincII restriction enzyme site (exon 2) and MseI restriction enzyme site (exon 5) are underlined. (B) Individual embryos injected with TALENs targeting lincRNAis18 exon 2 (left panel) or exon 5 (right panel). The presence of HincII and MseI digestion resistant amplicons demonstrates mutation of the targeted site. (TIF) [file pone.0114888.s007.tif]

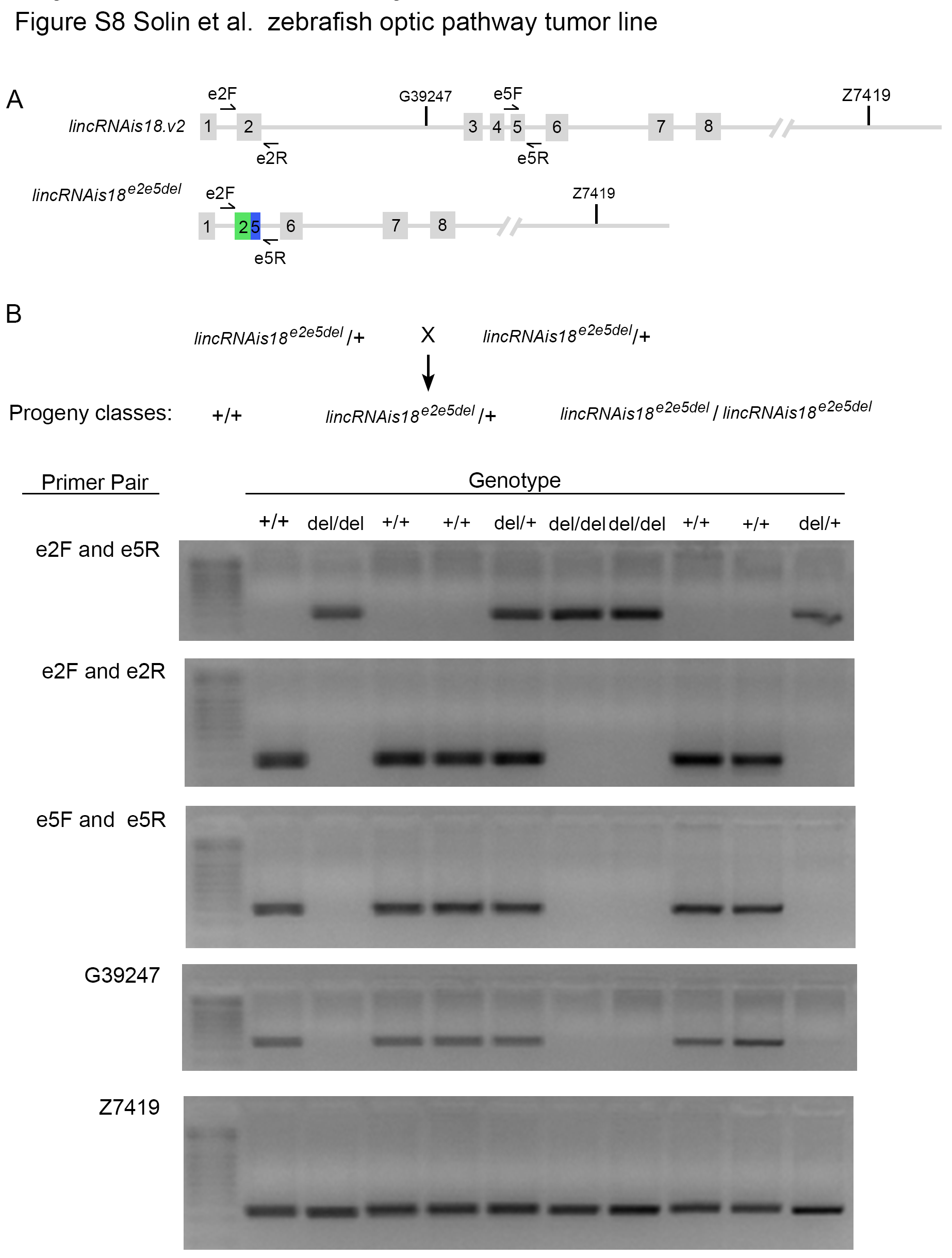

Supplement: S8 Figure — The lincRNAis18e2e5del deletion allele is homozygous viable. (A) Diagram of lincRNAis18 gene structure and exon 2- exon 5 deletion allele. Primers e2F and e2R flank exon 2; primers e5F and e5R amplify exon 5. The genetic marker G38247 is located between exons 2 and 3. The genetic marker Z7419 is located 1 Mb downstream of the 3′ end of lincRNAis18. (B) Genotyping of fin clips from 10 adult progeny of a lincRNAis18e2e5del/+ incross. Genomic DNA was amplified with primer pairs listed. The exon 2- exon 5 fusion amplicon was detected in 4/10 adults, indicating they were homozygous for the lincRNAis18e2e5del chromosome (top panel). As expected these 4 adults lacked the G39247 marker (4th panel). (TIF) [file pone.0114888.s008.tif]

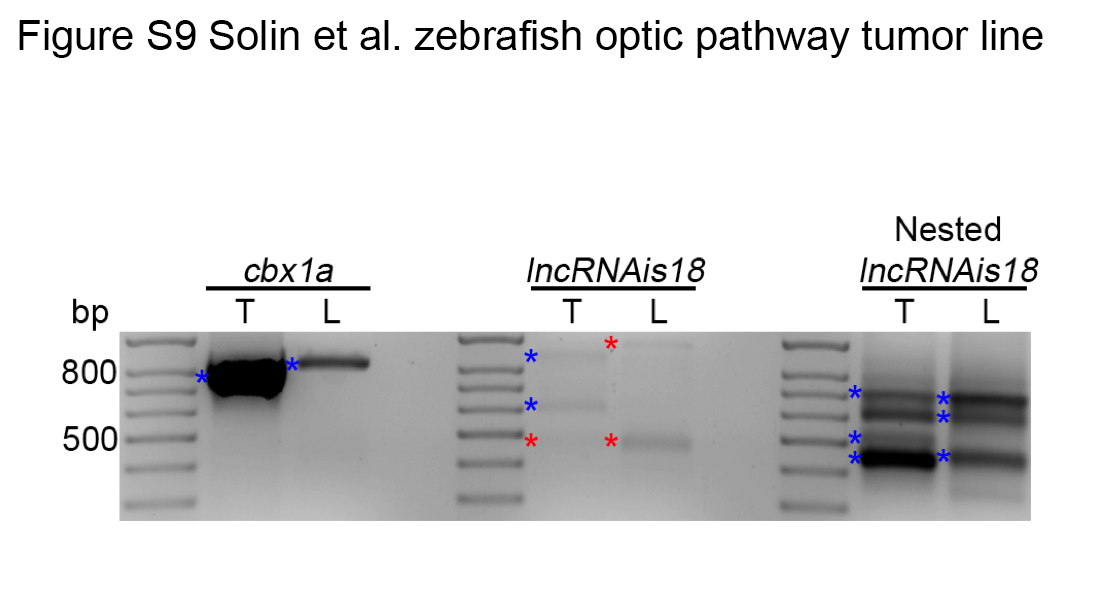

Supplement: S9 Figure — Loss of lincRNAis18 mRNA at polyA selection step during RNA-Seq library preparation. RT-PCR shows presence of cbx1a and lincRNAis18 in the total RNA from wild type retina sample used for RNA-Seq cDNA library preparation. The cbx1a transcript was detected following a single round of RT-PCR amplification in the total RNA sample (T) and in the Illumina RNA-Seq cDNA library sample (L). The lincRNAis18.v1 and.v2 transcripts were present in the total RNA sample (blue asterisks). However, only nonspecific products amplified from the cDNA library sample after one round of RT-PCR (red asterisks). Nested PCR resulted in amplification of multiple alternatively spliced transcripts from both samples (Right panel, blue asterisks). Bands marked with asterisks were cloned and sequence verified. (TIF) [file pone.0114888.s009.tif]
